# Unraveling the Role of the Microbiota in Cancer Immunotherapy: A New Frontier

**DOI:** 10.34133/research.0744

**Published:** 2025-06-24

**Authors:** Shouliang Wang, Shun Li, Mengli Zhang, Ruihan Liu, Xigang Ye, Siyi Mao, Jianwu Yu, Xinhua Xie, Weige Tan

**Affiliations:** ^1^ Guangzhou Medical University, Guangzhou 511436, China.; ^2^ Department of Breast Surgery, The First Affiliated Hospital, Guangzhou Medical University, Guangzhou 510120, China.; ^3^ State Key Laboratory of Oncology in South China, Guangdong Provincial Clinical Research Center for Cancer, Sun Yat-Sen University Cancer Center, Guangzhou 510060, China.

## Abstract

Cancer immunotherapy has greatly changed the therapeutic landscape for metastatic malignancies. Nevertheless, due to immune-related adverse events, drug resistance, and other factors, cancer immunotherapy remains largely untapped. Recent research has shown that the microbiota is crucial in shaping immune function and that its modulation can influence antitumor immunity. However, because of the intricate nature of the microbiome and immune system, a comprehensive mechanistic framework for understanding how the microbiota influences antitumor immune responses is still lacking. In this review, we summarize the mechanisms of the microbiota in antitumor immunity. We also comprehensively outline the methods for measuring the microbiota and their limitations. Additionally, we discuss the key challenges facing the targeting of the microbiota as a regulatory strategy for cancer immunotherapy.

## Introduction

The microbial communities, comprising viruses, archaea, bacteria, and eukaryotes that colonize the human body, are collectively referred to as the microbiota [1]. Over the past decade, extensive studies have linked the host microbiome to normal physiological processes and functions. The disruption of host microbial community homeostasis (known as dysbiosis) impacts the physiological equilibrium of human health, influencing various physiological systems, such as immunity, inflammation, digestion, and metabolism [2]. Importantly, alterations in the normal microbiota may also play a role in the onset of various diseases, such as cancer.

Cancer, a complex and multifactorial disease, is recognized as a primary cause of death worldwide [3,4]. Over the past decade, immunotherapy has been one of the most revolutionary developments in cancer treatment. Specifically, immune checkpoint blockade (ICB) therapy has markedly altered the therapeutic landscape for various solid tumors. Nonetheless, the complete potential of ICB remains unrealized due to the emergence of resistance and immune-related adverse events (irAEs) linked to treatment. Several studies have illustrated the impact of the microbiota on the modulation of antitumor immune responses. An individual's “baseline” microbiota is not only critical for immune homeostasis but may also influence tumor immunosurveillance and an individual's response to immune checkpoint inhibitors (ICIs).

In this review, we summarize the roles and mechanisms of the microbiota in antitumor immunity and the immune response. We provide a comprehensive summary of current methods for measuring the microbiota and their limitations. Finally, we discuss prospective strategies related to cancer immunotherapy that target microbiota regulation and the issues that need to be addressed.

## The Microbiota and Antitumor Immunity

The immune system has a complex and diverse relationship with tumor development, and it can monitor and control tumor growth through a series of complex mechanisms, thereby eradicating tumor cells. However, tumor cells can acquire the ability to escape immune system recognition, resulting in a failure of immune surveillance and ultimately tumor growth and progression [5]. Cancer immunoediting highlights the dual role of the immune system in controlling tumor proliferation and shaping tumor immunogenicity and describes 3 main steps of tumor development: elimination, equilibrium, and escape [6]. In the elimination phase, tumor cells are detected and eliminated by the host's immune system, while the residual tumor cells transition into the equilibrium phase. When tumor cells acquire resistance to immune responses, they escape. Several mechanisms have been identified as playing a role in the evolution of escape, including cytokine expression and the up-regulation of immune checkpoint proteins [7,8]. Cancer immunotherapy has been developed based on studies of tumor escape mechanisms, with the primary aims of reactivating the antitumor immune response and overcoming tumor cell escape. The early stage of cancer immunotherapy is based on the modulation of cytokines to affect immune cell function. For example, renal cell carcinoma can be treated with high doses of interleukin-2 (IL-2) [9,10]. The emergence of cancer immunotherapies, particularly those dominated by ICIs, has subsequently revolutionized cancer treatment in the last decade. ICB therapy has dramatically improved the survival rate of patients with multiple cancer types and has greatly changed the treatment outlook for metastatic malignancies[11–14]. Nevertheless, the complete therapeutic potential of ICB has not yet been achieved, as not all patients obtain long-term benefits, and a substantial proportion present initial resistance or develop resistance to therapy [15,16]. How to overcome resistance to ICIs and improve the immune response against tumors will be the next focus of cancer immunotherapy.

Heterogeneous treatment outcomes and the development of ICI resistance can be attributed to various factors, among which the gut microbiota plays a role in influencing the response to ICB across various cancer types [17,18]. For example, a substantial correlation has been observed between gut microbiota diversity and the response to anti-programmed death receptor 1 (PD-1) immunotherapy in patients with hepatobiliary cancers and melanoma [19–21]. Multiple preclinical and clinical cohort studies have shown that patients who respond to treatment have different gut microbiota "signatures", which are often linked to enhanced intratumoral immune cell infiltration and systemic immunity. Administering *Bifidobacterium* orally can increase dendritic cell (DC) activation, thereby enhancing CD8^+^ T-cell activation in the tumor microenvironment (TME) and facilitating anti-programmed death-ligand 1 (PD-L1) efficacy [22]. *Bifidobacterium fragilis* can activate antitumor T helper 1 (Th1) cells and increase the efficacy of anti-CTLA-4 therapy [23]. Several studies have shown that “responder” and “nonresponder” phenotypes can be recapitulated in antibiotic-treated mouse models or germ-free animals by fecal microbiota transplantation (FMT) [24,25]. Oral supplementation with *Akkermansia muciniphila* after FMT of nonresponder feces increased the recruitment of CCR9^+^CXCR3^+^CD4^+^ T lymphocytes to mouse tumors and restored the efficacy of PD-1 blockade [24]. Further prospective trials revealed that fecal levels of *A. muciniphila*, accompanied by a more inflamed TME, were associated with an increased objective response rate and overall survival, irrespective of PD-L1 expression, performance status, and antibiotics [26]. In addition, the gut microbiota can affect other forms of cancer immunotherapy. The clinical response of patients with B-cell malignancies receiving anti-CD19 chimeric antigen receptor (CAR)-T-cell therapy is related to specific bacterial taxa [27]. The elimination of gram-positive bacteria improved the efficacy of adoptive T-cell therapy (ATC) in mice with cervical cancer [28]. Overall, these findings indicate that the gut microbiota has great potential for improving cancer immunotherapy.

With the advancement of sequencing technology in the 21st century, organs or tissues that were once considered sterile (such as the breast, lung, and liver) have been reported to have an intratumoral microbiota. In 2020, extensive research on the microbiota within tumors provided evidence of the spatial distribution and intracellular localization of the microbiota in tumors and a novel category of microbial-based cancer diagnostics was proposed [29]. Two years later, 2 studies on the distribution of fungi in various tumors and their synergistic effects with bacteria pushed the study of the intratumoral microbiota to a new level of popularity [30,31]. Narunsky-Haziza et al. detected fungi intracellularly across 35 cancer types, with distinct community compositions specific to each cancer type (e.g., *Malassezia* in breast cancer and *Aspergillus* in lung cancer) [30]. Furthermore, there were substantial positive correlations between bacterial and fungal abundances, diversities, and co-occurrences. Three distinct mycobiome–bacteriome–immunome driven by fungal co-occurrence substantially distinguished immune response subtypes. Research has revealed that the probable origins of the intratumoral microbiota can be divided into 3 distinct types: (a) the intratumor microbiota, where microorganisms colonized in mucosal sources pass through the mucosal barrier due to mucosal damage; (b) the intratumor microbiota derived from adjacent normal tissues; and (c) the intratumor microbiota derived via the circulatory system, where microorganisms from the intestines, etc. diffuse via the bloodstream to tumor tissues [32]. Similar to the gut microbiota, the intratumoral microbiota can influence the development of the tumor immune microenvironment and antitumor immunity. In a pancreatic ductal adenocarcinoma (PDAC) mouse model, endogenous bacterial ablation not only promoted the differentiation of CD4^+^ T cells into Th1 cells and CD8^+^ T-cell activation but also up-regulated PD-1 expression, thereby increasing the efficacy of immunotherapy and preventing tumor growth [33]. The presence of microbiota within tumor tissue enhances Treg immunosuppression, thereby mediating the progression of prostate cancer and liver cancer [34,35].

The role of the microbiota (both gut and intratumoral) in antitumor immunity has been well documented; however, a consensus regarding the specific components of the microbiota associated with antitumor immunity is lacking [36,37]. In a large-scale metagenomic study, Routy et al. observed a correlation between the clinical response to ICIs and the relative abundance of *A. muciniphila*, whereas Gopalakrishnan et al. found that responders had a greater relative proportion of *Faecalibacterium prausnitzii* than nonresponders did [24,25]. In addition, a study involving 5 observational cohorts of patients with advanced cutaneous melanoma revealed a notable connection between the gut microbiome and the response to ICIs, which was dependent on the cohort. An analysis via machine learning corroborated the association between the microbiota and the overall response rate (ORR) to ICIs. However, microbiome-based features have shown limited reproducibility between different cohorts [38]. Although *A. muciniphila*, *Roseburia* spp., and *Bifidobacterium pseudocatenulatum* were linked to responders, no individual species could be considered a wholly consistent biomarker across studies. This problem is likely affected by multiple factors, such as DNA collection and extraction protocols, the variability in microbiome features among responders, the sample size, and statistical power issues. In summary, the role of the microbiome in the antitumor immune response appears to be more complicated than initially considered. Therefore, deeper insights into how the microbiome contributes to the antitumor immune response are necessary, and larger and more diverse standardized metadata and metagenomic data cohorts are needed to better explain the function of specific components of the microbiota in immunotherapy.

## The Biological Mechanism of Microbiota-Mediated Antitumor Immunity

### Signaling pathway

#### Pattern recognition receptor signaling

**Fig. 1.**
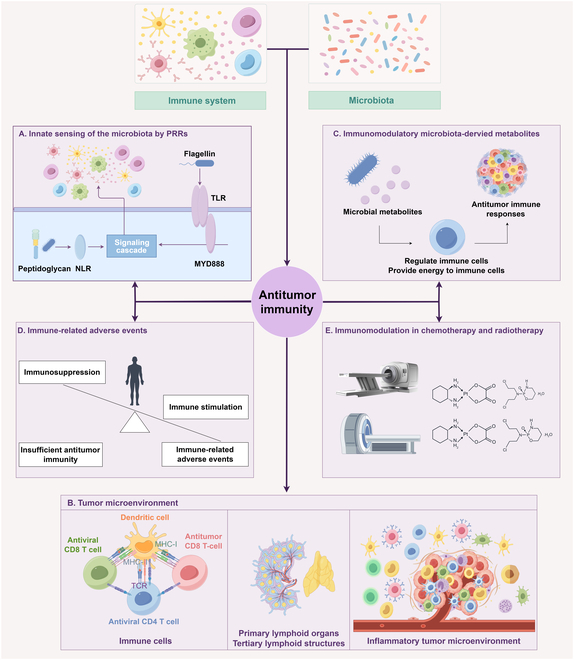
The biological mechanism of microbiota-mediated antitumor immunity. PRRs, pattern recognition receptors; NLR, nucleotide oligomerization domain (NOD)-like receptor; TLR, Toll-like receptor; Myd88, myeloid differentiation primary response 88; TCR, T-cell receptor; MHC, major histocompatibility complex.

Pattern recognition receptors (PRRs), which are regarded as the critical link between innate and adaptive immunity, are expressed primarily in innate immune cells [39]. PRRs can recognize microbe-associated molecular patterns (MAMPs) to initiate immune responses. Among PRRs, Toll-like receptors (TLRs) and nucleotide oligomerization domain (NOD)-like receptors (NLRs) are associated primarily with maintaining microbial homeostasis (Fig. 1A). TLRs are membrane-bound signaling receptors, and different subtypes recognize the membrane components or nucleic acids of pathogenic microorganisms in heterodimeric or homodimeric forms. TLR5 activates immune cells by recognizing flagellin of microorganisms, transforming the immunosuppressive TME into an immune-responsive state and thereby inhibiting tumor growth [40]. In contrast, research has indicated that TLR5-dependent commensal bacteria activating myeloid-derived suppressor cells (MDSCs) by increasing systemic IL-6 levels, thereby weakening antitumor immune responses and accelerating malignant progression [41]. These diverse findings could be associated with the polymorphic expression of TLR5 and its expression in different cancer tissues. For example, the TLR5R392X polymorphism can truncate the transmembrane signaling domain of TLR5, abolishing the flagellin response. In addition, the microbiota binds to TLRs on immune cells, which can induce an inflammatory, immunosuppressive environment and promote tumor progression. The expression of TLR4 (a receptor for gram-negative bacterial-derived lipopolysaccharide) is increased in colon cancer, inducing inflammation and promoting colon tumor progression [42]. TLR ligands derived from the lung microbiota can stimulate alveolar macrophages and neutrophils to produce IL-23 and IL-1β in a myeloid differentiation primary response 88 (Myd88)-dependent manner, increasing local inflammation and promoting lung cancer progression [43].

NLRs are intracellular PRRs that recognize pathogens and their components present in the cytoplasm. Among the NLR family members, NOD1 and NOD2 sense the peptidoglycan (PGN) component of microbial cell walls. NOD1 mainly recognizes γ-D-glucose-iso-diaminopimelic acid (iE-DAP), a component of the cell wall specific to gram-negative bacteria [44]. In addition to recognizing muramyl dipeptide within the walls of bacterial cells, NOD2 also recognizes complete viral single-stranded RNA (ssRNA). NODs exhibit remarkable activity within the intestinal environment and recognize the caspase recruitment domain (CARD–CARD). Through a CARD–CARD interaction, NODs form a complex with receptor-interacting protein 2 (RIP2) kinase to further trigger nuclear factor kappa-B (NF-κB) activation, thereby inducing inflammation [45]. Studies have shown that the activation of NOD2 can affect host immunity through multiple pathways (including the production and activation of CD8^+^ T cells, macrophages, and type 1 DCs) and improve the ICI response to eliminate tumor cells [46]. In conclusion, investigating the pattern recognition mechanism of PRRs is highly valuable for enhancing our understanding of how the microbiota regulates antitumor immunity.

#### STING signaling

Stimulator of interferon gene (STING), a cytosolic protein responsible for sensing DNA, is expressed in a range of cell types, including DCs, lymphocytes, and macrophages. Upon pathogen infection, cyclic GMP–AMP synthase (cGAS), a cytosolic PRR, recognizes pathogen-derived DNA and produces cyclic GMP–AMP (cGAMP), which then triggers the STING signaling pathway in host cells [47]. STING binds to cGAMP and up-regulates intracellular transcriptional programs, promoting the expression of type I interferons (IFNs) [48]. The administration of STING agonists, whether systemically or directly into the tumor, can reverse immunosuppression and promote tumor regression by inducing IFN production and activating immune cells [49]. Multiple studies have shown that the microbiota regulates STING signaling to improve the efficacy of cancer immunotherapy. The systemic administration of *Bifidobacterium* results in its accumulation in tumors, activating STING signaling in DCs and increasing the efficacy of anti-CD47 immunotherapy [50]. A combined treatment involving *L. rhamnosus* GG and anti-PD-1 shifted the gut microbial community toward the enrichment of *Bacteroides uniformis* and *Lactobacillus murinus*, inducing cGAS/STING-dependent IFN-I production, which increased CD8^+^ cell recruitment and DC activation in tumors [51]. Therefore, the microbiota shows promise in regulating the STING signaling pathway to reverse immunosuppression and improve the efficiency of immunotherapy by activating CD8^+^ T cells and DCs.

In addition, the microbiota is also capable of modulating antitumor immune responses through NF-κB signaling. The abundance of *Fusobacterium nucleatum* is substantially increased in colorectal adenomas and carcinomas, promoting tumor progression by producing NF-κB proinflammatory signaling and increasing the population of CD11b^+^ myeloid cells within the TME [52]. However, notably, the various signals do not exist independently but involve interactions between multiple components across different pathways. The specific components of the microbiota that regulate antitumor immune responses, the specific signaling pathways regulated by the microbiota, and the specific mechanisms of coordination between different signals still need to be addressed.

### Tumor microenvironment

The TME denotes the surrounding microenvironment in which tumor cells exist and comprises a diverse array of cellular and noncellular components. These components include surrounding immune cells, bone marrow-derived inflammatory cells, fibroblasts, blood vessels, the extracellular matrix and various signaling molecules. The importance of the TME in tumor growth, invasion, metastasis, and the therapeutic response is increasingly recognized, which has led to a shift in tumor research from a tumor-centric model to a model that views the TME as a whole. Recent studies have indicated that the microbiota changes the immune tone of the TME by regulating immune cells, primary and tertiary lymphoid structures (TLSs), and the inflammatory TME, thereby affecting the antitumor immune response [53–55] (Fig. 1B).

#### Immune cells

Cytotoxic CD8^+^ T cells, a type of killer lymphocyte, are a crucial part of the immune system and eliminate tumor cells within favorable tumor niches [56]. After priming, CD8^+^ T cells move and penetrate tumor locations under the guidance of chemokine and cytokine signaling to perform their antitumor functions. Upon the engagement of the T-cell receptor (TCR) on cytotoxic T cells with antigenic peptide–major histocompatibility complex (MHC)-I complexes, activated CD8^+^ T cells secrete granzyme A/B, perforin, and IFN-γ to induce tumor cell necrosis and pyroptosis. Therefore, a greater number of infiltrating CD8^+^ T cells is often positively associated with antitumor immune responses. Specific components of the microbiota (intestinal microbiota Clostridiales strains, *L. rhamnosus* GG, intratumoral *Lachnoclostridium*, etc.) have been shown to activate CD8^+^ T cells, thereby enhancing antitumor immunity [54,57–59]. Dysbiosis of the microbiota can overstimulate CD8^+^ T cells, leading to their premature exhaustion, thereby reducing antitumor immunity, and thus increasing tumor susceptibility [60]. Notably, compared with ICI treatment, Montalban-Arques et al. found that supplementation with specific Clostridiales bacterial species had better efficacy at increasing CD8^+^ T-cell infiltration [54]. Clostridiales supplementation was also effective in experimental lung cancer, breast cancer and melanoma models, suggesting a broad-spectrum antitumor mechanism associated with this therapeutic approach [54]. In general, identifying specific components of the microbiota that increase CD8^+^ T-cell infiltration could represent a promising therapeutic strategy for overcoming resistance to cancer immunotherapy.

CD4^+^ T cells are highly versatile and multifunctional cells that recognize and bind to exogenous antigenic peptides presented by MHC class II molecules and differentiate into several different functional subtypes (e.g., Th1, Th2, and Th17) in response to different signals. Previously, neoantigen vaccines were shown to stimulate multifunctional CD4^+^ T cells to differentiate into CD4^+^ Th1 cells in preclinical mouse tumor models or clinical trials with limited sample sizes, thereby inducing a prolonged CD8^+^ T-cell response [61–63]. In contrast, immunization with exact-length MHC I-restricted peptides (which specifically target CD8^+^ T cells) elicited only a fleeting CD8^+^ T-cell response. With increasing research on the microbial regulation of CD4^+^ T-cell production, *Helicobacter hepaticus* (Hhep)-induced antitumor immunity was not observed to depend on CD8^+^ T cells but on CD4^+^ T cells [55]. Oral supplementation with *A. muciniphila* increased the infiltration of CCR9^+^CXCR3^+^CD4^+^ T cells into murine tumors and restored the efficacy of PD-1 blockade [24]. These findings suggest that CD4^+^ T cells not only support antitumor CD8^+^ T-cell responses but also independently contribute to antitumor immunity. Interestingly, Zhu et al.[57] observed that patients with cutaneous melanoma who had low CD4^+^ T cell counts presented substantially greater survival rates than did those with high CD4^+^ T cell counts. This result is likely because immunosuppression is a characteristic feature of human cancers, causing CD4^+^ T cells to differentiate into iTregs rather than Th1 cells. Therefore, in-depth investigations into how the microbiota correctly regulates CD4^+^ T-cell differentiation into the desired functional subtypes is critical.

DCs, a diverse class of specific antigen-presenting cells, are involved in innate and adaptive immunity and are essential for antitumor immune responses [64]. The microbiota has been reported to affect the phenotype and function of DCs. Bacteria such as *A. muciniphila*, *Bifidobacterium*, and *Bacteroides fragilis*, along with their related metabolic products, can activate DCs [23,28,65]. Activated DCs reverse the immune tolerance induced by immature DCs and stimulate T-cell activation. Introducing a stable model microbiota into germ-free mice can restore DC function and initiate T-cell responses [65]. For example, oral *Bifidobacterium* administration enhances CD8^+^ T-cell responses in the TME by activating DCs via the TLR4 pathway [22]. Vancomycin-treated intestinal microbiota improve antitumor-specific effector T-cell activity by increasing DC and IL-12 levels [28].

Natural killer (NK) cells are cytotoxic innate lymphocytes that regulate the levels of DCs and CD8^+^ T cells within the TME [66]. Jin et al. demonstrated that non-small cell lung cancer (NSCLC) patients exhibiting high gut microbial diversity presented an increased abundance of NK cell subsets in the periphery in response to PD-1 blockade, which suggests that the microbiota may play a regulatory role in NK cell activation [19]. More evidence of interactions between the microbiota and NK cells has subsequently emerged. *Lactobacillus plantarum* activates NK cells to induce innate immunity by increasing natural cytotoxicity receptor protein expression [67]. A high-salt diet increases the abundance and intratumoral localization of *Bifidobacteria*, which increases NK cell activation and promotes melanoma regression [68].

#### Primary lymphoid organs and TLS

Primary lymphoid organs (also referred to as central immune organs) serve as sites for the generation, differentiation, development, and maturation of immune cells, including those in the bone marrow and thymus. The translocation of the intestinal microbiota exacerbates preleukemic bone marrow dysplasia, ultimately resulting in precursor B-cell acute lymphoblastic leukemia [69]. The high diversity of the intestinal microbiota can substantially increase the effectiveness of allogeneic hematopoietic stem cell transplantation in leukemia patients [70]. These studies suggest that the microbiota may be related to bone marrow function. A subsequent joint analysis of daily fluctuations in patients' differential blood cell counts and longitudinal microbiota samples revealed a close relationship between the intestinal microbiota composition and immune reconstitution dynamics [71]. This effect may be partially attributed to the delivery of endogenous ligands for retinoic acid-inducible gene I (RIG-I), including 3pRNA and RNA from bacteria, phages, or viruses, which can trigger IFN-I signaling for protection [72].

TLSs are aberrant formations of lymphoid tissue in nonlymphoid organs that resemble secondary lymphoid organs and occur during chronic inflammation or within tumor environments. TLS maturation promotes lymphocyte activation and infiltration within tumor tissues, playing a vital role in antitumor immunity. Hhep induces the production of follicular helper T cells, enhances Hhep^+^ tumor-adjacent TLS maturation, increases the tumor infiltration of cytotoxic lymphocytes, and thereby suppresses tumor growth [55]. These results indicate that the tumor microbiota is crucial for TLS-derived antitumor immunity.

#### Inflammatory TME

Inflammation is the body's defense response to external damaging factors (such as infection, trauma, and chemicals) or endogenous stimuli (such as autoimmune reactions). The activation of acute inflammatory responses stimulates the maturation and antigen presentation of immune cells such as DCs, thereby exerting antitumor immune effects. Chronic, dysregulated, persistent, and unresolved inflammation can form the inflammatory TME for tumor growth in various tissues and is an important factor in tumor development [73]. Numerous studies have indicated that the disruption of the host–microbiota boundary activates PRRs, leading to the production of various cytokines and the activation of the NF-κB signaling pathway. NF-κB acts as the primary regulator of cancer-associated inflammation, facilitating a chronic inflammatory positive feedback loop that promotes carcinogenesis in nontumor cells and induces the transformation of tumor cells [73]. Symbiotic bacteria increase local inflammation in lung cancer by increasing γδ T-cell activation and the production of proinflammatory factors such as IL-17, thereby leading to tumor progression [43]. *F. nucleatum* adheres to colorectal cancer cells via its unique adhesin FadA, stimulating the release of inflammatory factors (such as NF-κB) and facilitating colorectal tumor growth [74]. In addition, *F. nucleatum* is involved in the formation of an immunosuppressive environment, thereby facilitating tumor initiation, progression, and metastasis. *F. nucleatum* inhibits antitumor immunity by binding to tyrosine-based inhibitory motif (TIGIT) and impairing the tumor cell-killing functions of NK cells and T cells [75]. Controlling tumor-related chronic inflammation represents a promising avenue for antitumor therapy. For example, the *L. plantarum* strain YYC-3 exerts anticancer effects by down-regulating inflammatory cytokine expression and inhibiting the activation of the NF-κB and Wnt signaling pathways [76]. Thus, a comprehensive understanding of how dysregulated inflammation, tumor progression, and microbiota-regulated inflammation are interconnected is necessary for the development of novel antitumor strategies.

### Microbial metabolites

#### Short-chain fatty acids

Short-chain fatty acids (SCFAs) are fatty acids generated by intestinal microorganisms through the fermentation of unabsorbed dietary fiber and other carbohydrates and mainly include acetic acid, propionate, and butyrate. Recently, the correlation between SCFAs and PD-1 inhibitor efficacy in treating malignancies has been substantiated [77]. As research progresses, multiple mechanisms have been suggested to underlie the relationship between SCFAs and ICI responses. First, SCFAs inhibit tumor cell growth and induce apoptosis. Butyrate, a metabolite of *Holdemanella biformis* and *Faecalibaculum rodentium*, increases protein acetylation by inhibiting calcineurin/NFATc3 activation, thereby preventing tumor cell proliferation [78]. Second, SCFAs can enhance the antitumor immune response. Research has shown that SCFAs can directly increase the antitumor cytotoxicity of CD8^+^ T cells. Butyrate enhances tumor-specific CD8^+^ T-cell cytotoxicity in an ID2-dependent manner by activating the IL-12 signaling pathway. Furthermore, it facilitates effector CD8^+^ T-cell differentiation with memory capacity and improved recall ability [79,80]. In addition, butyrate and valerate can enhance the function of mTOR as a sensor of central cellular metabolism, leading to increased production of effector molecules and thereby substantially promoting the antitumor effects of CAR T cells and antigen-specific cytotoxic T lymphocytes (CTLs) [81]. Third, SCFAs improve the efficacy of ICIs by providing energy to immune cells. SCFAs provide energy for antibody production and B-cell differentiation through cellular metabolism by regulating energy metabolism to increase glycolysis, oxidative phosphorylation, and fatty acid synthesis [82].

However, controversy and contradictions remain regarding the functions of SCFAs in the therapeutic effects of ICIs. In an analysis of the gut microbial metabolome of NSCLC patients treated with nivolumab, patients with propionate and butyrate enrichment in their feces experienced better therapeutic effects [83]. Similarly, high fecal SCFA concentrations are associated with therapeutic responsiveness and longer progression-free survival of patients with various malignancies who are treated with anti-PD-1 antibodies [77]. Conversely, elevated blood butyrate levels restrict DCs and tumor-specific T cells, thus inhibiting the antitumor activity of patients with metastatic melanoma who are treated with anti-CTLA-4 antibodies [84]. This discrepancy may depend on the therapeutic modality and tissue compartment. Thus, further research is needed to clarify the exact roles of SCFAs and their potential as markers of ICI efficacy.

#### Inosine

Inosine is a purine metabolite generated by *Bifidobacterium pseudolongum* and *A. muciniphila* and is involved in energy metabolism, signal transduction, and nucleic acid synthesis in organisms [85]. Recent studies have indicated that inosine can regulate immune cell metabolism and improve responses to ICI therapy. The high metabolic requirements of cancer cells restrict effector T-cell function by depleting nutrients and generating immunosuppressive metabolites. Under energy restriction (glucose deficiency), inosine can supply biosynthetic precursors and ATP for the pentose phosphate and glycolytic pathways, providing an alternative carbon source to support effector CD8^+^ T-cell production [86]. Combination therapy with PD-L1 antibodies and inosine outperforms PD-L1 antibody monotherapy in reducing tumor growth and prolonging overall survival. However, the antitumor efficacy of ICIs combined with inosine requires costimulators, such as IL-12 and CpG. The inosine–A2AR–cAMP–PKA signaling cascade facilitates cAMP response element-binding protein phosphorylation, leading to the up-regulation of IFN-γ and IL-12Rβ2 transcription. This process promotes Th1 cell differentiation and accumulation within the TME [87]. In addition, inosine can also increase tumor immunogenicity. Inosine increases tumor antigen levels by directly suppressing the ubiquitin-activating enzyme UBA6 in tumor cells, facilitating the recognition and destruction of tumor cells by cytotoxic immune cells [88].

#### Bile acid and tryptophan metabolites

Secondary bile acids are produced from primary bile acids in the gut through bacterial metabolism. Secondary bile acids exert immunosuppressive effects. For example, isolithocholic acid and 3-oxolithocholic acid suppress Th17 cell differentiation by inhibiting the key Th17 transcription factor retinoic acid receptor-related orphan nuclear receptor γt [89]. 3β-Hydroxydeoxycholic acid acts on DCs, promotes naive CD4^+^ T-cell differentiation into regulatory T cells, and leads to immune escape [90]. Colonization with bile acid-metabolizing bacteria or supplementation with secondary bile acids can activate liver CXCR6^+^ NKT cells, reversing liver tumor growth inhibition [91].

Tryptophan is another metabolite associated with immunosuppression. Microbiota-driven tryptophan catabolism generates aryl hydrocarbon receptor (AhR) ligands, such as kynurenine. Kynurenine facilitates the differentiation of naive CD4^+^ T cells into Foxp3^+^ regulatory T cells through AhR activation, resulting in immunosuppression [92]. A higher kynurenine/tryptophan ratio is correlated with shorter overall survival of melanoma and renal cell carcinoma patients receiving anti-PD-1 therapy [93]. Similarly, elevated activity of indoleamine-2,3-dioxygenase 1, an essential enzyme in the tryptophan-to-kynurenine pathway, is associated with primary resistance to immunotherapy in NSCLC [94]. 5-Hydroxytryptophan (5-HTP), another tryptophan catabolite, induces CD8^+^ T-cell exhaustion via STAT5–5-HTP–AhR axis activation, resulting in immunosuppression [95].

#### Vitamin B family, trimethylamine N-oxide

With advancements in research, more microbial metabolites have been identified as influential in antitumor immune responses. A meta-analysis of ICI responders and nonresponders revealed that vitamin B family metabolic pathways are enriched in patients with metastatic melanoma who responded to ICI therapy [96]. The microbial metabolite trimethylamine N-oxide (TMAO) enhances CD8^+^ T-cell-mediated antitumor immunity by inducing pyroptosis in triple-negative breast cancer (TNBC) [97]. Additionally, TMAO increases type I IFN signaling by promoting an immunostimulatory phenotype in macrophages, thereby generating antitumor effects [98].

Overall, microbial metabolites are important bridges in the complicated balance between the microbiota and host immune activity, substantially influencing the therapeutic outcomes of antitumor immunity (Fig. 1C and Table 1). Future studies incorporating metabolomic and transcriptomic analyses of the microbiota may connect metabolites with microbial gene expression and function, aiding in the recognition of clinical biomarkers and therapeutic targets for specific cancers.

**Table 1.** Immunoregulatory mechanisms of microbial metabolites

Microbial metabolites	Targeted pathway	Functions	References
Butyrate	Activates the IL-12 signaling pathway	Enhances chemotherapy efficacy through CD8^+^ T-cell-mediated antitumor immune responses	[80]
Butyrate	Down-regulates tumor-specific and memory T cell accumulation	Diminishes the antitumor efficacy of CTLA-4 blockade	[84]
Butyrate	Modulates cytotoxic CD8 T-cell receptor signaling	Promotes the antitumor efficacy of anti-PD-1 treatment	[157]
Butyrate	Activates cytotoxic CD8^+^ T cells	Boosts anti-PD-1 efficacy	[158]
Propionate butyrate	Induce DAMP release and promote DC maturation	Delay the progression of acute myeloid leukemia	[159]
Pentanoate butyrate	Inhibit class I histone deacetylase activity	Enhance the antitumor activity of CTLs and CAR T cells	[81]
Acetate butyrate	Induce cancer cell autophagy and M2 macrophage polarization	Promote prostate cancer progression	[160]
Acetate	Bolsters T-cell effector functions and proliferation	Potentiates antitumor immunity	[161]
Short-chain fatty acids	Increase acetyl-CoA levels	Promote B-cell differentiation into antibody-producing cells	[82]
Short-chain fatty acids	Increase cellular metabolism	Boost CD8^+^ T-cell effector functions	[162]
Short-chain fatty acids	Induce DNA damage and activate cGAS/STING signaling	Activate antitumor immunity	[163]
Inosine	Inhibits UBA6 in tumor cells	Augments tumor immunogenicity and responses	[88]
3-Oxolithocholic acid	Inhibits retinoic acid receptor-related orphan nuclear receptor-γt	Suppresses Th17 cell differentiation	[89]
5-Hydroxytryptophan	Activates aryl hydrocarbon receptor nuclear translocation	Cause T cell dysfunction in the tumor microenvironment	[95]
Deoxycholic acid	Inhibits Ca2^+^-NFAT2 signaling	Suppresses CD8^+^ T-cell responses	[164]
Kynurenine	Promotes naive CD4^+^ T-cell differentiation into foxp3 regulatory T cells by activating aryl hydrocarbon receptor	Immunosuppression	[92]
Indoles	Activate the aryl hydrocarbon receptor in tumor-associated macrophages	Suppress antitumor immunity	[165]
Indole-3-aldehyde	Activates the aryl hydrocarbon receptor	Enhances antitumor immune responses and improves ICI efficacy	[166]
Indole-3-propionic acid	Increases H3K27 acetylation at the superenhancer region of Tcf7	Promotes the differentiation of CD8^+^ T cells into progenitor exhausted CD8^+^ T cells	[167]
Trimethylamine N-oxide	Activate endoplasmic reticulum stress kinase PERK	Enhance CD8^+^ T-cell-mediated antitumor immunity	[97]
Trimethylamine N-oxide	Potentiates the type I interferon pathway	Relieves immunosuppression in the tumor microenvironment of pancreatic cancer	[98]

### Immune-related adverse events

With advances in immunotherapy, an increasing number of ICIs are now being used to treat specific cancers. However, not all patients benefit from ICI treatment, and a considerable number of patients suffer from irAEs. These irAEs are characterized by various toxicities, including hepatitis, colitis, rash, myocarditis, and pneumonia, which result from immune dysregulation during the administration of immunotherapies such as ICIs. Usyk et al. [99] found that the 1-year risk of irAEs differed nearly sevenfold between patients with high abundances of *Bacteroides vulgatus* and *Bacteroides dorei*. These findings suggest that the microbiota composition can influence the toxicity associated with immunotherapy. Studies have also revealed an association between an increased abundance of *Bacteroides intestinalis* and intestinal toxicity in melanoma patients [100]. *Bifidobacterium* increases the immunosuppressive capacity of Tregs via IL-10 regulation, alleviating intestinal immunopathology during anti-CTLA-4 immunotherapy without substantially impacting antitumor immunity [101,102]. Overall, the microbiota influences both the local and systemic immune tone, thereby affecting the balance between immune stimulation and immune regulation (Fig. 1D). This balance between immune stimulation and regulation likely underpins both the irAE response and development. Identifying components of the microbiota that can reduce the incidence of toxicity or treat irAEs is a promising therapeutic strategy.

### Immunomodulation in chemotherapy and radiotherapy

Chemotherapy and radiotherapy are traditional cancer treatments that reduce or eliminate the tumor burden mainly by directly killing tumor cells (Fig. 1E). Recent studies have revealed that chemotherapy and radiotherapy induce the release-specific signaling molecules during tumor cell death, activating tumor-specific immune responses and inducing abscopal effects [103–105]. Cyclophosphamide, a commonly used anticancer drug, exerts therapeutic effects partly by stimulating antitumor immune responses. Viaud et al. [106] showed that cyclophosphamide affects the gut microbiota composition and promotes the translocation of specific bacteria to secondary lymphoid organs, stimulating Th17 and Th1 immune responses. Vancomycin treatment eliminates the response to cyclophosphamide treatment. Additionally, SCFAs enhance CD8^+^ T-cell responses in an ID2-dependent manner, thus increasing oxaliplatin efficacy [80]. In contrast, Uribe-Herranz et al.[104] found that vancomycin treatment eliminates SCFA-producing bacteria, enhances antigen presentation, promotes cytotoxic T-cell infiltration, and improves the efficacy of local radiotherapy. A possible explanation for the differing results with vancomycin treatment lies in the distinct mechanisms of the 2 models. Another plausible explanation is that localized radiotherapy predominantly circumvents direct impacts on the intestinal environment, whereas chemotherapy, a systemic intervention, induces alterations in the intestinal microbiota. As previous studies have shown, total body irradiation directly impacts the intestinal microbiota and damages the intestinal epithelial barrier, thereby affecting the tumor response [107]. Overall, further research is needed to understand how the microbiota influences immunomodulation.

## Techniques for Assessing and Measuring the Microbiota

The modulatory effects of the microbiota offer many possibilities for clinical intervention. The microbiome composition could serve as a complementary predictive or prognostic biomarker for treatment outcomes. Multiple studies have indicated that specific components of the microbiota are substantially associated with the expression of immunoregulatory genes, immune cell infiltration, and the levels of inflammatory factors, indicating potential predictive value for ICI efficacy. An accurate microbiota assessment and measurement could provide targeted guidance for cancer immunotherapy.

Traditionally, the assessment of clinical microbiomes involves the collection of samples from the target site, followed by the cultivation of organisms and subsequent phenotypic and molecular characterization. This process is notably time-consuming and labor-intensive. Furthermore, the cultivation of microorganisms can potentially obscure the microbial abundance in the original sample due to selective or exclusive growth. Recent methodological advances have made cell-free methods for measuring microbial presence and activity increasingly popular. 16S ribosomal RNA (rRNA) gene sequencing is a widely used technique for profiling and classifying complex bacterial communities. The 16S rRNA gene encodes rRNA in prokaryotes and contains both highly conserved and 9 hypervariable regions (V1 to V9). Hypervariable regions are useful for identifying and classifying species [108]. Specifically, this approach involves employing a strategy that utilizes universal primers targeting conserved sequences around hypervariable regions to create an amplicon library for sequencing. Sequences are clustered into amplicon sequence variants (ASVs) or operational taxonomic units (OTUs) based on similarity for taxonomic annotation [109]. Because sequencing targets a segment of the 16S rRNA gene in prokaryotes, its resolution is limited to the genus level and excludes nonbacterial microorganisms such as fungi and viruses. The development of a new 5R multiplex sequencing approach and the application of biotinylated 16S primer strategies can substantially increase the coverage and accuracy of 16S rRNA sequencing for tumor-resident bacteria [29,110]. Moreover, because most bacteria have multiple 16S rRNA gene copies, the relative abundance can be artificially elevated, leading to an inaccurate quantification of bacterial cells. Although the technique has limitations, its affordability and high-throughput capability make it the most frequently employed method for an initial descriptive analysis.

Unlike 16S rRNA gene sequencing, shotgun metagenomic sequencing indiscriminately sequences all the genomes in a sample, covering bacteria, archaea, fungi, and viruses. This method enhances species and strain-level distinction by providing multiple marker gene sequences, enabling the computational reconstruction of the microbial community composition and facilitating the identification of new microbial genomes from unnamed species [111]. However, in tumors rich in host cells and DNA, obtaining the sequencing depth required to detect sufficient microbial reads may result in higher costs (e.g., added computing power, data storage, and complex pipelines), and the accuracy of the results may also be influenced by contamination with human genomic DNA. To mitigate the challenge of overwhelming host DNA, several host–DNA depletion strategies have been developed, including enzymatic digestion (e.g., DNase treatment following selective lysis), chemical depletion methods, and hybridization-based capture of human sequences. While these methods can substantially enrich microbial DNA, they may introduce bias by preferentially depleting certain taxa or failing to remove intracellular host DNA. Additionally, shotgun metagenomic sequencing can elucidate the functional capabilities of the microbiota using gene prediction tools and databases. Given an adequate sequencing depth, shotgun metagenomic sequencing enables the inference of microbial functional information for the entire community and facilitates the annotation of genes associated with antibiotic resistance, metabolic pathways, or toxin production through comparisons with established databases [112]. In summary, when strain-level resolution, low-abundance detection, non-bacterial taxa, or functional profiling is required, shotgun metagenomic sequencing is more suitable. Optimizing the sequencing protocol to balance sequencing depth, cost, and host DNA removal efficiency is critical to maximize the utility of shotgun metagenomic data in tumors.

Single-cell RNA sequencing (scRNA-seq) is a revolutionary technology for quantifying the expression of genes at the individual cell level, enabling the generation of comprehensive gene expression profiles for many cells. While microbial scRNA-seq is recognized as valuable, the application of scRNA-seq technology to microorganisms has been hampered by the low bacterial mRNA content, the absence of polyadenylation in bacterial mRNA, and so on. High-throughput microbial scRNA-seq techniques, including combinatorial barcoding strategies and scDual-Seq, which capture both pathogen and host transcriptomes simultaneously, have enhanced the assessment of gene expression in prokaryotes [113–115]. However, these methods fail to maintain the spatial context of the analyzed cells, limiting their effectiveness in analyzing single-species and multispecies biofilms. With the development of high phylogenetic resolution spatial transcriptomics using fluorescence in situ hybridization (HiPRFISH) and parallel sequential fluorescence in situ hybridization (parseqFISH), visualizing the interactions between host cells and microbial communities has become increasingly possible [116,117]. Imaging-based spatial transcriptomics not only provides a comprehensive analysis of gene expression levels by traditional transcriptomics but also retains the spatial location information of cells in tissues, thereby revealing the characteristics of gene expression between different cell types and detecting changes in tissues under various physiological or pathological conditions [118]. Galeano Niño et al. revealed spatial, cellular, and molecular host–microbe interactions by combining in situ spatial analysis technology with scRNA-seq, thus laying a foundation for investigating the spatial transcriptomes of host–microbiome interactions within the TME [119].

Nongenomic microbiome characterization refers to methods for analyzing microbial communities and their functions without relying on direct genome sequencing or nucleic acid analysis. This characterization approach focuses on studying microbial communities from the perspective of functionality, metabolites, proteins, and other nongenetic materials, including metabolomics, MHC peptidomics, and multicondition microbiome cultivation (“culturomics”). Metabolomics is a method for detecting and identifying small-molecule metabolites through high-throughput analytical technology and is used to characterize thousands of potential bioactive metabolites produced or regulated by diverse components of the microbiota [77]. SCFAs can help stratify progression-free survival outcomes via metabolomics of patients with tumors receiving anti-PD-1 therapy. MHC peptidomics involves the study of peptides (i.e., short-chain amino acid fragments) that interact with MHC molecules. These peptides can be presented on the cell surface by MHC molecules, allowing them to interact with T cells and potentially induce an immune response. Recently, research has shown that peptides produced from intracellular bacteria can be presented by tumor cells, thereby activating immune responses [120]. These findings provide insights into how bacteria may influence immune activation and therapeutic outcomes. Culturomics enhances the culturability of microorganisms by replicating diverse environmental conditions, allowing for the precise classification and functional assessment of individual bacteria within a microbial ecosystem [121].

**Fig. 2.**
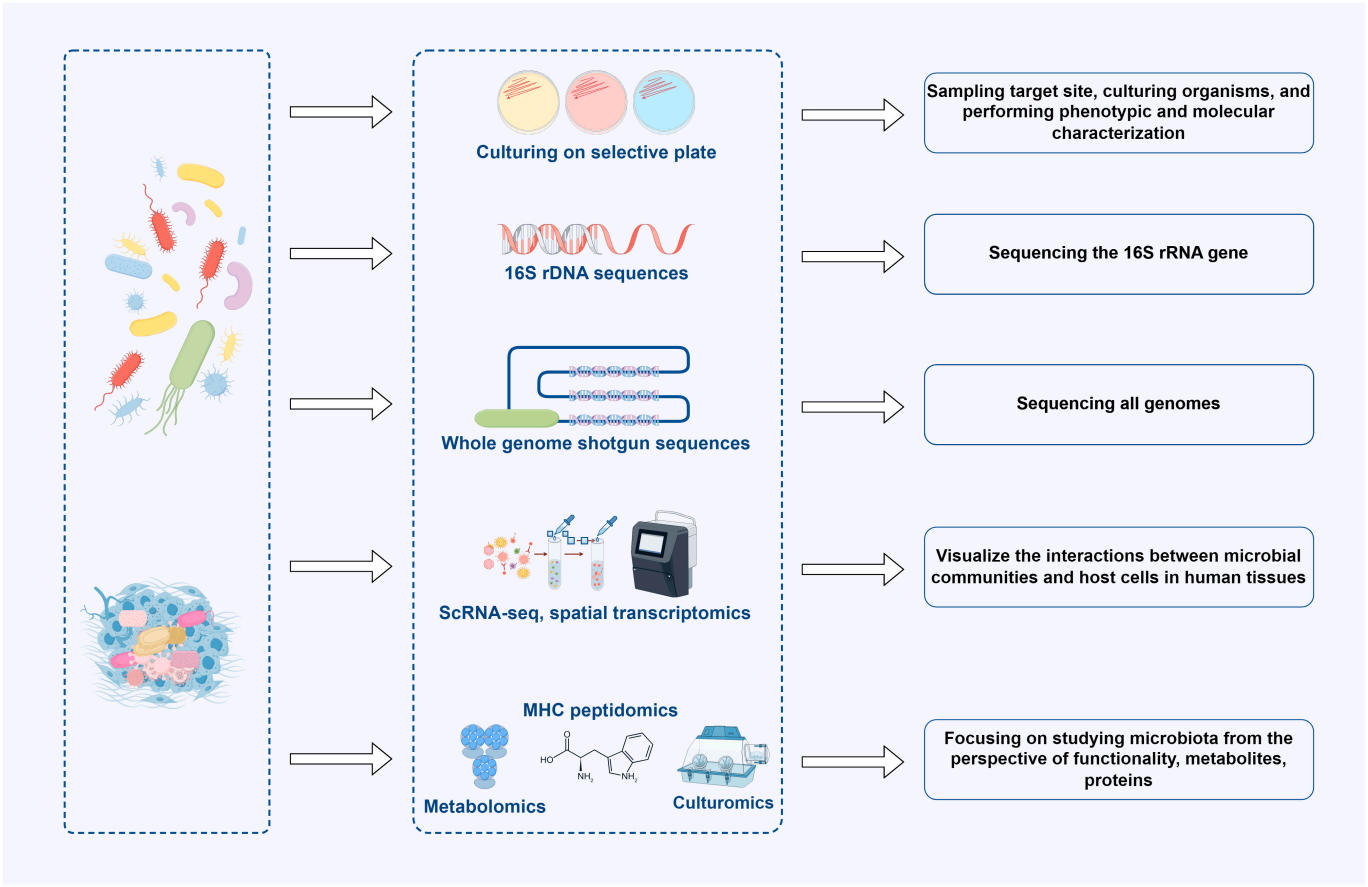
Technologies for assessing and measuring the microbiota.

In summary, the measurement of the microbiota facilitates both qualitative and quantitative evaluations of the microbial community composition, function, and metabolic activities through the application of diverse omics technologies and analytical methodologies (Fig. 2). Although current microbiota measurement methods have made remarkable progress, they still have their own limitations due to contamination, low sensitivity, technical limitations, etc. Accurate measurement of the microbiota remains a challenge.

## Strategies for Regulating the Microbiota

**Fig. 3.**
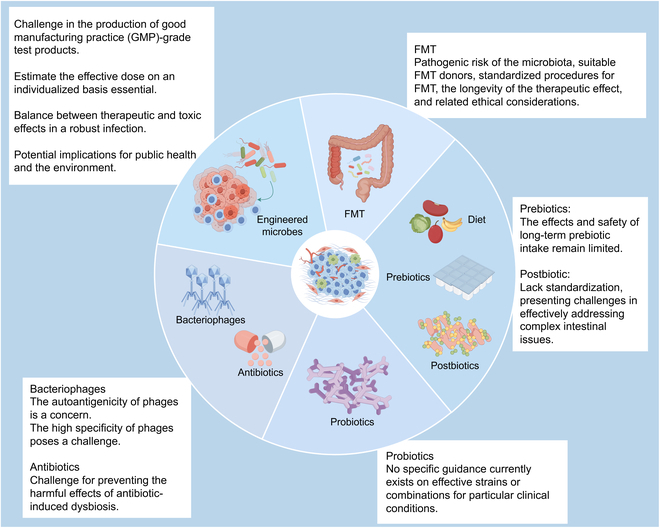
Problems and limitations of microbiota modulation. FMT, fecal microbiota transplantation.

Currently, multiple preclinical studies indicate that microbiota regulation enhances ICI activity. Considering the crucial influence of the microbiome on modulating the immune response, strategies aimed at regulating or reconstructing the microbiome to bolster antitumor activity while minimizing the incidence of severe irAEs are becoming a central focus in the advancement of future cancer therapies. Given the role of the microbiome in shaping immune responses, this section discusses current and prospective strategies for targeting microbiome regulation in cancer immunotherapy (Fig. 3).

### Fecal microbiota transplantation

FMT is an early strategy for regulating microbiota that includes transplanting feces from a specific donor to the recipient. FMT is frequently employed to treat *Clostridium difficile* infection and ulcerative colitis, with efficacy in the relief of clinical symptoms and a well-established safety profile. A phase I clinical trial of patients with advanced melanoma revealed that FMT in patients who did not respond to ICB therapy could effectively reverse previous resistance[122,123]. In addition, multiple phase I and phase II clinical trials are underway to investigate the efficacy of FMT in treating various solid tumors (Table 2). Despite the promising preliminary outcomes observed in patients treated with FMT, researchers must exercise caution in its application. Prior to the routine implementation of FMT, several clinical, regulatory, and scientific uncertainties need to be resolved, including the pathogenic risk of the microbiota, suitable FMT donors, standardized procedures for FMT, the longevity of the therapeutic effect, and related ethical considerations.

**Table 2.** Clinical studies on microbiome manipulation in cancer immunotherapy

Intervention	Treatment combination	Cancer entity	Study phase	Enrollment	Status	Objective	Primary outcome	Trial identifier
FMT	Immune checkpoint inhibitors	Malignancy	Not applicable	18	Completed	Investigation of FMT for patients with malignant tumors who do not respond to cancer immunotherapy	Change in the intestinal microbiome community	NCT05273255
FMT	Standard (chemo-)immunotherapy	Metastatic lung cancer	Phase 2	80	Recruiting	Examines the safety and efficacy of FMT in conjunction with immunotherapy	Progression-free survival rate	NCT05502913
FMT	Immune checkpoint inhibitors	Metastatic melanoma	Phase 2	24	Recruiting	Investigation of whether FMT from either responder or nonresponder patients can alter the response to immunotherapy	Stable disease, partial response, and complete response	NCT05251389
FMT	Ipilimumab and nivolumab	Renal cell carcinoma	Phase 1	20	Active, not recruiting	Examines the safety and efficacy of FMT combination treatment	Occurrence of immune-related colitis	NCT04163289
FMT	Atezolizumab or bevacizumab	Advanced hepatocellular carcinoma	Phase 2	48	Not yet recruiting	Examines the safety of FMT combined with immunotherapy	Differential tumoral CD8 T-cell infiltration and adverse event documentation	NCT05690048
FMT	Keytruda	Metastatic mesothelioma	Early phase 1	1	Completed	Examines the safety and efficacy of FMT combined with Keytruda	Progression-free survival rate	NCT04056026
FMT	PD-1 or PD-L1 inhibitors	Advanced lung cancer	Not applicable	25	Completed	Examines the safety of FMT combined with immunotherapy	Safety	NCT04924374
FMT	Immune checkpoint blockade	Advanced melanoma	Phase 2	128	Not yet recruiting	Assesses the efficacy of LND101 for FMT in combination with ICB	Progression-free survival rate	NCT06623461
FMT	Immune checkpoint inhibitors	Cancer patients not responding to ICI therapy	Phase 2	20	Recruiting	Assesses the safety and efficacy of FMT combination with ICI therapy	Safety evaluation and tumor response evaluation	NCT05286294
FMT	Tislelizumab, pemetrexed, and platinum	Advanced non-small cell lung cancer	Phase 2	62	Not yet recruiting	Assesses the safety and efficacy of FMT/immunotherapy/chemotherapy	12-months progression-free survival rate	NCT06403111
FMT	Sintilimab	Advanced gastric cancer	Phase 2	198	Not yet recruiting	Examines the safety and efficacy of FMT combined with SOX and sintilimab	2-year overall survival rate	NCT06405113
FMT	Pembrolizumab or nivolumab	Melanoma	Phase 1	20	Active, not recruiting	Examines the effects of FMT in combination with immunotherapy	Safety	NCT03772899
FMT	PD-1 or PD-L1 inhibitors	Gastrointestinal cancer	Phase 1	10	Completed	Determines whether the FMT capsule can help reverse the resistance to anti-PD-(L)1 treatment	Objective response rate, adverse events, and rate of abnormal vital signs and laboratory test results	NCT04130763
FMT	Pembrolizumab or nivolumab	Metastatic colorectal cancer	Phase 2	15	Active, not recruiting	Evaluates the efficacy of pembrolizumab or nivolumab in conjunction with FMT	Objective response rate	NCT04729322
FMT	Atezolizumab and bevacizumab	Advanced hepatocellular carcinoma	Phase 2	12	Recruiting	Evaluates the safety and efficacy of FMT combined with atezolizumab plus bevacizumab	Safety	NCT05750030
FMT	Immune checkpoint inhibitors	Renal cell carcinoma	Phase 2	50	Active, not recruiting	Evaluates the efficacy of targeted FMT in improving response rates to ICIs	Progression-free survival rate	NCT04758507
FMT	5-FU pumping combined with immunotherapy	Metastatic nasopharyngeal carcinoma	Phase 3	96	Not yet recruiting	Assesses PFLL combined with a PD-1 antibody with or without FMT	Progression-free survival rate	NCT06486220
Diet	Anti-PD-1 and anti-CTLA4 immunotherapy	Metastatic melanoma	Not applicable	30	Recruiting	Evaluates the effects of an individualized nutritive intervention based on the Mediterranean diet on the changes in the immunotherapy response	Change in the level of ingested flavones, anthocyanins, vitamin D, omega-3 fatty acids, and fiber	NCT06236360
Prebiotic food-enriched diet	Immune checkpoint blockade	Unresectable melanoma	Phase 2	75	Recruiting	Evaluates the effects of a prebiotic food-enriched diet on patients with melanoma who are starting ICB therapy	Change in *Faecalibacterium*	NCT06466434
High-fiber diet	Anti-PD1, anti-CTLA4, and/or anti-LAG3 monoclonal antibodies	Advanced melanoma	Not applicable	40	Recruiting	Assesses the effects of a high-fiber diet on immunotherapy outcomes	Change in gut microbiome diversity	NCT06298734
Diet	Pembrolizumab or nivolumab	Stage III–IV melanoma	Phase 2	50	Active, not recruiting	Evaluates the effect of the diet on the immune system	Change in the gut microbiome	NCT04645680

### Diet, prebiotics, and postbiotics

Nutritional interventions represent a promising approach to modulate the microbiota because of their cost-effectiveness, favorable safety, and noninvasive nature. Dietary modifications can enhance the beneficial microbiota and modify the immune–microbiome landscape. Studies indicate that transitioning from animal-based to plant-based diets can quickly modify the gut microbiota and related metabolites [124,125]. For cancer patients, higher fiber intake, more fiber-fermenting microorganisms, and greater microbial diversity have all been associated with the response to ICIs [126,127]. A ketogenic (low-carbohydrate, moderate-protein, and high-fat) diet has also been shown to enhance the antitumor activity of anti-PD-1 therapy through the major ketone body 3-hydroxybutyrate (3HB) [128]. However, this intervention is more likely to benefit individuals who previously did not achieve the recommended intake levels than the entire population. Additionally, because rapid alterations in the gut microbiota and metabolism are easily reversed, sustained dietary changes are essential to achieve long-term health benefits. The swift alterations over a brief time frame highlight the practicality of short-term dietary changes before or during ICI treatment, which provides a possibility to enhance the treatment effects and reduce susceptibility to toxicity.

Dietary supplementation with specific prebiotics (e.g., inulin and pectin) represents an additional strategy to modulate the microbiota by selectively supporting the growth of beneficial bacteria in the intestine, thereby indirectly improving the intestinal flora balance. Therefore, this method might primarily increase the growth of certain microbial groups instead of increasing the overall microbial diversity. Inulin, a polysaccharide dietary fiber, effectively modulates the gut microbiota to induce memory T-cell responses, substantially increasing the efficacy of anti-PD-1 therapy in a mouse adenocarcinoma model [129]. A pectin-rich diet that induces type I IFN production within the TME increases the efficacy of anti-PD-1 therapy [130]. However, most studies have focused on animals, ignoring “real-life” interindividual differences in human physiology and disease-related responses. Prebiotics rely on the existing flora in the intestine. If the intestinal flora is insufficient or absent, the efficacy of prebiotics may be substantially diminished. Furthermore, clinical studies examining the effects and safety of long-term prebiotic intake remain limited. Therefore, understanding individual and response variability and ensuring treatment safety have become a priority for further research.

Postbiotic therapy is a treatment modality that utilizes metabolites or secretions produced by probiotics to increase host health. Unlike probiotics, postbiotics do not require live strains to remain active; instead, they directly exert effects. For example, elevated levels of mevalonic acid and dimethylglycine in the intestines of germ-free mice are associated with improved antitumor immunity and increased ICI efficacy [59]. Cell-free supernatants derived from *Faecalibacterium* spp., *Bifidobacterium*, and *Lactobacillus* activate anti-inflammatory signaling pathways and reduce tumor cell proliferation [131]. In addition, another postbiotic approach involves the use of outer membrane vesicles (OMVs) as delivery vehicles for tumor-modulating cargo. Antigen-modified OMVs can present various tumor antigens, thereby eliciting a synergistic antitumor immune response that effectively eliminates melanoma metastasis and inhibits subcutaneous colorectal cancer growth [132]. However, the mechanisms of postbiotics remain incompletely understood, and their targeting is less precise than that of probiotics. In addition, postbiotics lack standardization, presenting challenges in effectively addressing complex intestinal issues. Consequently, randomized clinical trials are needed to establish the postbiotic supplementation frequency and optimal dose for cancer patients.

### Probiotics

Probiotics are live microbes that benefit the host by colonizing the body and modifying the host's microbiota composition at specific sites. Unlike dietary or prebiotic interventions, probiotics directly introduce specific microorganisms to the host. Probiotic preparations are considered to influence cancer pathogenesis and antitumor immunity, but conflicting results have been produced in multiple cases. Specific intestinal bacteria (such as *B. fragilis*, *L. rhamnosus* GG, *Lacticaseibacillus paracasei*, *A. muciniphila*, *Bifidobacterium*, or probiotic combinations) can enhance ICI efficacy in mouse models [21,23,24,51,59,133]. A phase I trial showed that CBM588, a bifidogenic live bacterial product, improved the efficacy of checkpoint inhibitors in patients with metastatic renal cell carcinoma receiving nivolumab–ipilimumab therapy [134]. In contrast, some studies indicate that probiotic supplements may have limited or no benefit for cancer patients and could even cause adverse reactions. For example, over-the-counter probiotics containing *L. rhamnosus* GG or *Bifidobacterium longum* reduced ICI effectiveness in patients with advanced melanoma [127]. These inconsistent results may result from individual differences in colonization resistance to external probiotics, which are influenced by the native microbiota [135]. Additionally, due to the heterogeneity of probiotic strains and the limited number of studies on the effects of multiple probiotic strains under specific criteria, no specific guidance currently exists on effective strains or combinations for particular clinical conditions.

With the advancement of computer predictions of new bioactive "precision probiotics" that rely on multiomics (such as genomics, transcriptomics, metabolomics, and proteomics) and the management of specific cultivated species microbiota, the development of new “precision probiotic” preparations that are safe, have high colonization ability, and produce an efficient antitumor immune response is now possible [136]. Ongoing clinical trials are investigating the efficacy of combining selected components of the microbiota and ICIs in treating advanced cancer (Table 2). Additionally, synbiotics, which combine prebiotics and probiotics, may be more effective than prebiotics alone, as they can increase microbial diversity and overcome the limitations of single-strain probiotics.

### Antibiotics and bacteriophages

In addition to introducing beneficial bacteria, eliminating pathogenic or harmful bacteria within the microbiota is also a potential strategy. Antibiotics reduce microbial diversity, altering the composition of the microbiota [137]. The antibiotic-induced eradication of cancer-associated microorganisms can prevent gastric cancer by eliminating *Helicobacter pylori* [138,139]. Antibiotics inhibit the occurrence of colon tumors by eliminating carcinogenic bacteria. However, the nonspecificity of most antibiotics also consumes beneficial commensal bacteria and affects antitumor immune responses. Studies have indicated that cancer patients receiving ICI therapy who have previously received antibiotics exhibit an increased incidence of irAEs and markedly lower objective response and overall survival rates [140–142]. In general, antibiotics are ineffective at enhancing ICI efficacy and should be avoided whenever possible. However, in some cases, antibiotics are often used as life-saving treatments for cancer patients with infections. Therefore, methods to prevent the harmful effects of antibiotic-induced dysbiosis on ICI efficacy are important topics of research. For example, studies have shown that combining DAV132 (a colon-targeted adsorbent) with antibiotics can preserve microbiome diversity and maintain anti-PD-1 antibody activity [143,144].

Bacteriophages (phages) are viruses that specifically target and destroy bacteria, representing the most abundant members of the enterovirome. Unlike traditional antibiotics, phage-based therapies have garnered attention for their capacity to selectively target highly resistant microorganisms while preserving microbiota homeostasis. Engineered *F. nucleatum*-specific phages have been developed to deliver irinotecan nanoparticles, effectively eradicating *F. nucleatum* and mitigating the adverse effects associated with nonspecific drug delivery to healthy tissues [145]. Phage-based cancer immunotherapy remains in its early stages and must overcome several limitations before it can be used in clinical practice. First, the autoantigenicity of phages is a concern. Phages, as foreign organisms, trigger immunological responses that may diminish therapeutic efficacy and lead to immune cell exhaustion. Second, the high specificity of phages poses a challenge. Phages have remarkable specificity and generally infect only specific bacterial strains. Bacteria can resist phage infection by altering surface receptors, changing cell membrane structures, etc. Once bacteria acquire resistance to phages, the therapeutic effect is substantially reduced.

### Targeting tumors: Engineered microbes

In addition to the oral administration of whole microbiome configurations or isolated probiotics, recent experimental studies have explored cancer treatment through the introduction or modification of intratumoral microbes. Tumor-targeting bacteria can serve as delivery vehicles to improve drug targeting specificity and reduce patient toxicity while also providing metabolic support for intratumoral T cells and overcoming the immunologically “cold” TME. Currently, *Escherichia coli* Nissle (EcN), *Lactobacillus*, and *Salmonella typhimurium* are being used to develop engineered bacterial cancer therapies [146–149]. For example, engineered EcN strains have been designed to express single-domain antibody fragments targeting PD-L1 and CTLA-4 within tumors, thereby augmenting antitumor immunity [150]. The modification of EcN can convert ammonia into L-arginine, supplying essential metabolites for cytotoxic T-cell-mediated antitumor immunity [151,152]. Furthermore, various oncolytic microbial agents have shown promising antitumor efficacy in many cancer settings. These agents include oncolytic viruses that selectively target and destroy cancer cells and bacteria such as *Clostridium novyi*-NT, which are modified to enhance therapeutic outcomes by removing toxin-producing genes. The oncolytic virus talimogene laherparepvec (T-VEC) with pembrolizumab achieved a 30% ORR in sarcoma patients [153]. An intratumoral injection of the adenovirus DNX-2401 induces direct oncolysis and subsequent immune-mediated antiglioma responses in patients with recurrent malignant glioma [154]. An intratumoral injection of *C. novyi*-NT can reduce the tumor burden and enhance tumor-specific T-cell responses in patients with advanced tumors [155].

Numerous challenges in clinical application necessitate further exploration to alleviate the systemic inflammatory immune response and cytokine release syndrome triggered by microorganisms and tumor lysis and acknowledge the distinctive characteristics of live engineered microorganisms as therapeutic agents [156]. First, unlike inactive substances, live engineered microorganisms cannot be sterilized through conventional means, presenting a grand challenge in the production of good manufacturing practice (GMP)-grade test products. Second, the effective dose of live engineered microorganisms depends on factors such as the degree of tumor necrosis and the abundance of existing tumor-infiltrating inflammatory cells, making an estimation of the effective dose on an individualized basis essential. Third, oncolytic microbial therapy aims to intentionally convert tumors into localized tumor-destructive infections. Early antibiotic action may eradicate the infection prior to the manifestation of the antitumor effect, whereas a delayed intervention poses the possibility of unanticipated systemic inflammatory responses, highlighting the importance of achieving a careful balance. Finally, the clinical application of live biological agents inevitably has potential implications for public health and the environment, necessitating a careful consideration of appropriate management strategies.

## Conclusions

The study of the microbiota in relation to cancer has rapidly progressed in recent years. Extensive research indicates that the microbiota is crucial for modulating immune function and can affect antitumor immunity. Understanding the composition, manipulation, and intervention of the microbiota has become essential for comprehending cancer progression and immunotherapy. As research advances, more details about the intricate relationships among the host immune system, microbiota, cancer progression, and therapeutic interventions are emerging. Evolving tools and techniques for microbiota characterization, along with regulatory strategies, provide more data and broader treatment options at a lower cost. However, due to the complexity of the microbiome and immune system, coupled with the emerging status of this field, a comprehensive mechanistic framework for understanding how the microbiota influences antitumor immune responses is lacking. For example, what type of microbiota is most ideal, which populations benefit most, and what are the optimal strategies for microbiota regulation? Does the microbiota become established in the TME at the onset of tumorigenesis, or is it recruited following alterations in the microenvironment by the tumor? Is it possible to simultaneously enhance antitumor immunity and limit immune-mediated toxicity? Once these questions are solved, the microbiota will play a transformative role in cancer immunotherapy.

## References

[1] Singh RP, Bashir H, Kumar R. Emerging role of microbiota in immunomodulation and cancer immunotherapy. Semin Cancer Biol. 2021;70:37–52. 32580024 10.1016/j.semcancer.2020.06.008

[2] Dominguez-Bello MG, Godoy-Vitorino F, Knight R, Blaser MJ. Role of the microbiome in human development. Gut. 2019;68(6):1108–1114. 30670574 10.1136/gutjnl-2018-317503 PMC6580755

[3] Bray F, Laversanne M, Weiderpass E, Soerjomataram I. The ever-increasing importance of cancer as a leading cause of premature death worldwide. Cancer. 2021;127(16):3029–3030. 34086348 10.1002/cncr.33587

[4] Bray F, Laversanne M, Sung H, Ferlay J, Siegel RL, Soerjomataram I, Jemal A. Global cancer statistics 2022: GLOBOCAN estimates of incidence and mortality worldwide for 36 cancers in 185 countries. CA Cancer J Clin. 2024;74(3):229–263. 38572751 10.3322/caac.21834

[5] Kennedy LB, Salama AKS. A review of cancer immunotherapy toxicity. CA Cancer J Clin. 2020;70(2):86–104. 31944278 10.3322/caac.21596

[6] Dunn GP, Old LJ, Schreiber RD. The three Es of cancer immunoediting. Annu Rev Immunol. 2004;22:329–360. 15032581 10.1146/annurev.immunol.22.012703.104803

[7] Tumeh PC, Harview CL, Yearley JH, Shintaku IP, Taylor EJM, Robert L, Chmielowski B, Spasic M, Henry G, Ciobanu V, et al. PD-1 blockade induces responses by inhibiting adaptive immune resistance. Nature. 2014;515(7528):568–571. 25428505 10.1038/nature13954 PMC4246418

[8] Bhatia A, Kumar Y. Cellular and molecular mechanisms in cancer immune escape: A comprehensive review. Expert Rev Clin Immunol. 2014;10(1):41–62. 24325346 10.1586/1744666X.2014.865519

[9] Krieg C, Létourneau S, Pantaleo G, Boyman O. Improved IL-2 immunotherapy by selective stimulation of IL-2 receptors on lymphocytes and endothelial cells. Proc Natl Acad Sci USA. 2010;107(26):11906–11911. 20547866 10.1073/pnas.1002569107 PMC2900642

[10] Klapper JA, Downey SG, Smith FO, Yang JC, Hughes MS, Kammula US, Sherry RM, Royal RE, Steinberg SM, Rosenberg S. High-dose interleukin-2 for the treatment of metastatic renal cell carcinoma : A retrospective analysis of response and survival in patients treated in the surgery branch at the National Cancer Institute between 1986 and 2006. Cancer. 2008;113(2):293–301. 18457330 10.1002/cncr.23552 PMC3486432

[11] Overman MJ, Lonardi S, Wong KYM, Lenz HJ, Gelsomino F, Aglietta M, Morse MA, van Cutsem E, McDermott R, Hill A, et al. Durable clinical benefit with nivolumab plus ipilimumab in DNA mismatch repair-deficient/microsatellite instability-high metastatic colorectal cancer. J Clin Oncol. 2018;36(8):773–779. 29355075 10.1200/JCO.2017.76.9901

[12] Reck M, Rodríguez-Abreu D, Robinson AG, Hui R, Csőszi T, Fülöp A, Gottfried M, Peled N, Tafreshi A, Cuffe S, et al. Pembrolizumab versus chemotherapy for PD-L1-positive non-small-cell lung cancer. N Engl J Med. 2016;375(19):1823–1833. 27718847 10.1056/NEJMoa1606774

[13] Rini BI, Plimack ER, Stus V, Gafanov R, Hawkins R, Nosov D, Pouliot F, Alekseev B, Soulières D, Melichar B, et al. Pembrolizumab plus axitinib versus sunitinib for advanced renal-cell carcinoma. N Engl J Med. 2019;380(12):1116–1127. 30779529 10.1056/NEJMoa1816714

[14] Weber JS, D'Angelo SP, Minor D, Hodi FS, Gutzmer R, Neyns B, Hoeller C, Khushalani NI, Miller WH Jr, Lao CD, et al. Nivolumab versus chemotherapy in patients with advanced melanoma who progressed after anti-CTLA-4 treatment (CheckMate 037): A randomised, controlled, open-label, phase 3 trial. Lancet Oncol. 2015;16(4):375–384. 25795410 10.1016/S1470-2045(15)70076-8

[15] Ribas A, Wolchok JD. Cancer immunotherapy using checkpoint blockade. Science. 2018;359(6382):1350–1355. 29567705 10.1126/science.aar4060 PMC7391259

[16] Sharma P, Hu-Lieskovan S, Wargo JA, Ribas A. Primary, adaptive, and acquired resistance to cancer immunotherapy. Cell. 2017;168(4):707–723. 28187290 10.1016/j.cell.2017.01.017 PMC5391692

[17] Xie J, Liu M, Deng X, Tang Y, Zheng S, Ou X, Tang H, Xie X, Wu M, Zou Y. Gut microbiota reshapes cancer immunotherapy efficacy: Mechanisms and therapeutic strategies. iMeta. 2024;3: Article e156. 38868510 10.1002/imt2.156 PMC10989143

[18] Xia L, Zhu X, Wang Y, Lu S. The gut microbiota improves the efficacy of immune-checkpoint inhibitor immunotherapy against tumors: From association to cause and effect. Cancer Lett. 2024;598: Article 217123. 39033797 10.1016/j.canlet.2024.217123

[19] Jin Y, Dong H, Xia L, Yang Y, Zhu Y, Shen Y, Zheng H, Yao C, Wang Y, Lu S. The diversity of gut microbiome is associated with favorable responses to anti-programmed death 1 immunotherapy in Chinese patients with NSCLC. J Thorac Oncol. 2019;14(8):1378–1389. 31026576 10.1016/j.jtho.2019.04.007

[20] Mao J, Wang D, Long J, Yang X, Lin J, Song Y, Xie F, Xun Z, Wang Y, Wang Y, et al. Gut microbiome is associated with the clinical response to anti-PD-1 based immunotherapy in hepatobiliary cancers. J Immunother Cancer. 2021;9(12): Article e003334. 34873013 10.1136/jitc-2021-003334 PMC8650503

[21] Matson V, Fessler J, Bao R, Chongsuwat T, Zha Y, Alegre ML, Luke JJ, Gajewski TF. The commensal microbiome is associated with anti-PD-1 efficacy in metastatic melanoma patients. Science. 2018;359(6371):104–108. 29302014 10.1126/science.aao3290 PMC6707353

[22] Sivan A, Corrales L, Hubert N, Williams JB, Aquino-Michaels K, Earley ZM, Benyamin FW, Man Lei Y, Jabri B, Alegre ML, et al. Commensal Bifidobacterium promotes antitumor immunity and facilitates anti-PD-L1 efficacy. Science. 2015;350(6264):1084–1089. 26541606 10.1126/science.aac4255 PMC4873287

[23] Vétizou M, Pitt JM, Daillère R, Lepage P, Waldschmitt N, Flament C, Rusakiewicz S, Routy B, Roberti MP, Duong CPM, et al. Anticancer immunotherapy by CTLA-4 blockade relies on the gut microbiota. Science. 2015;350(6264):1079–1084. 26541610 10.1126/science.aad1329 PMC4721659

[24] Routy B, Le Chatelier E, Derosa L, Duong CPM, Alou MT, Daillere R, Fluckiger A, Messaoudene M, Rauber C, Roberti MP, et al. Gut microbiome influences efficacy of PD-1-based immunotherapy against epithelial tumors. Science. 2018;359(6371):91–97. 29097494 10.1126/science.aan3706

[25] Gopalakrishnan V, Spencer CN, Nezi L, Reuben A, Andrews MC, Karpinets TV, Prieto PA, Vicente D, Hoffman K, Wei SC, et al. Gut microbiome modulates response to anti-PD-1 immunotherapy in melanoma patients. Science. 2018;359(6371):97–103. 29097493 10.1126/science.aan4236 PMC5827966

[26] Derosa L, Routy B, Thomas AM, Iebba V, Zalcman G, Friard S, Mazieres J, Audigier-Valette C, Moro-Sibilot D, Goldwasser F, et al. Intestinal *Akkermansia muciniphila* predicts clinical response to PD-1 blockade in patients with advanced non-small-cell lung cancer. Nat Med. 2022;28(2):315–324. 35115705 10.1038/s41591-021-01655-5 PMC9330544

[27] Smith M, Dai A, Ghilardi G, Amelsberg KV, Devlin SM, Pajarillo R, Slingerland JB, Beghi S, Herrera PS, Giardina P, et al. Gut microbiome correlates of response and toxicity following anti-CD19 CAR T cell therapy. Nat Med. 2022;28(4):713–723. 35288695 10.1038/s41591-022-01702-9 PMC9434490

[28] Uribe-Herranz M, Bittinger K, Rafail S, Guedan S, Pierini S, Tanes C, Ganetsky A, Morgan MA, Gill S, Tanyi JL, et al. Gut microbiota modulates adoptive cell therapy via CD8α dendritic cells and IL-12. JCI Insight. 2018;3(4): Article e94952. 29467322 10.1172/jci.insight.94952 PMC5916241

[29] Nejman D, Livyatan I, Fuks G, Gavert N, Zwang Y, Geller LT, Rotter-Maskowitz A, Weiser R, Mallel G, Gigi E, et al. The human tumor microbiome is composed of tumor type-specific intracellular bacteria. Science. 2020;368(6494):973–980. 32467386 10.1126/science.aay9189 PMC7757858

[30] Narunsky-Haziza L, Sepich-Poore GD, Livyatan I, Asraf O, Martino C, Nejman D, Gavert N, Stajich JE, Amit G, González A, et al. Pan-cancer analyses reveal cancer-type-specific fungal ecologies and bacteriome interactions. Cell. 2022;185(20):3789–3806. 36179670 10.1016/j.cell.2022.09.005 PMC9567272

[31] Dohlman AB, Klug J, Mesko M, Gao IH, Lipkin SM, Shen X, Iliev ID. A pan-cancer mycobiome analysis reveals fungal involvement in gastrointestinal and lung tumors. Cell. 2022;185(20):3807–3822. 36179671 10.1016/j.cell.2022.09.015 PMC9564002

[32] Xie Y, Xie F, Zhou X, Zhang L, Yang B, Huang J, Wang F, Yan H, Zeng L, Zhang L, et al. Microbiota in tumors: From understanding to application. Adv Sci. 2022;9(21): Article e2200470. 10.1002/advs.202200470 PMC9313476 35603968

[33] Pushalkar S, Hundeyin M, Daley D, Zambirinis CP, Kurz E, Mishra A, Mohan N, Aykut B, Usyk M, Torres LE, et al. The pancreatic cancer microbiome promotes oncogenesis by induction of innate and adaptive immune suppression. Cancer Discov. 2018;8(4):403–416. 29567829 10.1158/2159-8290.CD-17-1134 PMC6225783

[34] Gao Y, You M, Fu J, Tian M, Zhong X, du C, Hong Z, Zhu Z, Liu J, Markowitz GJ, et al. Intratumoral stem-like CCR4^+^ regulatory T cells orchestrate the immunosuppressive microenvironment in HCC associated with hepatitis B. J Hepatol. 2022;76(1):148–159. 34689996 10.1016/j.jhep.2021.08.029

[35] Ma J, Gnanasekar A, Lee A, Li WT, Haas M, Wang-Rodriguez J, Chang EY, Rajasekaran M, Ongkeko WM. Influence of Intratumor microbiome on clinical outcome and immune processes in prostate cancer. Cancers. 2020;12(9):2524. 32899474 10.3390/cancers12092524 PMC7564876

[36] Zou Y, Zhang H, Liu F, Chen Z, Tang H. Intratumoral microbiota in orchestrating cancer immunotherapy response. J Transl Intern Med. 2024;12(6):540–542. 10.1515/jtim-2024-0038 PMC11720933 39802449

[37] Tang Y, Cai Q, Tian Z, Chen W, Tang H. Crosstalk between gut microbiota and cancer immunotherapy: Present investigations and future perspective. Research. 2025;8:600. 10.34133/research.0600 PMC11754537 39850365

[38] Lee KA, Thomas AM, Bolte LA, Björk JR, de Ruijter LK, Armanini F, Asnicar F, Blanco-Miguez A, Board R, Calbet-Llopart N, et al. Cross-cohort gut microbiome associations with immune checkpoint inhibitor response in advanced melanoma. Nat Med. 2022;28(3):535–544. 35228751 10.1038/s41591-022-01695-5 PMC8938272

[39] Li D, Wu M. Pattern recognition receptors in health and diseases. Signal Transduct Target Ther. 2021;6:291. 34344870 10.1038/s41392-021-00687-0 PMC8333067

[40] Chen J, Qiao Y, Chen G, Chang C, Dong H, Tang B, Cheng X, Liu X, Hua Z. *Salmonella flagella* confer anti-tumor immunological effect via activating Flagellin/TLR5 signalling within tumor microenvironment. Acta Pharm Sin B. 2021;11(10):3165–3177. 34729307 10.1016/j.apsb.2021.04.019 PMC8546927

[41] Rutkowski MR, Stephen TL, Svoronos N, Allegrezza MJ, Tesone AJ, Perales-Puchalt A, Brencicova E, Escovar-Fadul X, Nguyen JM, Cadungog MG, et al. Microbially driven TLR5-dependent signaling governs distal malignant progression through tumor-promoting inflammation. Cancer Cell. 2015;27(1):27–40. 25533336 10.1016/j.ccell.2014.11.009 PMC4293269

[42] Pastille E, Faßnacht T, Adamczyk A, Ngo Thi Phuong N, Buer J, Westendorf AM. Inhibition of TLR4 signaling impedes tumor growth in colitis-associated colon cancer. Front Immunol. 2021;12: Article 669747. 34025672 10.3389/fimmu.2021.669747 PMC8138317

[43] Jin C, Lagoudas GK, Zhao C, Bullman S, Bhutkar A, Hu B, Ameh S, Sandel D, Liang XS, Mazzilli S, et al. Commensal microbiota promote lung cancer development via γδ T cells. Cell. 2019;176(5):998–1013. 30712876 10.1016/j.cell.2018.12.040 PMC6691977

[44] Lavelle EC, Murphy C, O'Neill LAJ, Creagh EM. The role of TLRs, NLRs, and RLRs in mucosal innate immunity and homeostasis. Mucosal Immunol. 2010;3(1):17–28. 19890268 10.1038/mi.2009.124 PMC3428627

[45] Dai Z, Zhang J, Wu Q, Fang H, Shi C, Li Z, Lin C, Tang D, Wang D. Intestinal microbiota: A new force in cancer immunotherapy. Cell Commun Signal. 2020;18(1):90. 32522267 10.1186/s12964-020-00599-6 PMC7288675

[46] Netea MG, Domínguez-Andrés J, Barreiro LB, Chavakis T, Divangahi M, Fuchs E, Joosten LAB, van der Meer JWM, Mhlanga MM, Mulder WJM, et al. Defining trained immunity and its role in health and disease. Nat Rev Immunol. 2020;20(6):375–388. 32132681 10.1038/s41577-020-0285-6 PMC7186935

[47] Dvorkin S, Cambier S, Volkman HE, Stetson DB. New frontiers in the cGAS-STING intracellular DNA-sensing pathway. Immunity. 2024;57(4):718–730. 38599167 10.1016/j.immuni.2024.02.019 PMC11013568

[48] Sun L, Wu J, Du F, Chen X, Chen ZJ. Cyclic GMP-AMP synthase is a cytosolic DNA sensor that activates the type I interferon pathway. Science. 2013;339(6121):786–791. 23258413 10.1126/science.1232458 PMC3863629

[49] Jing W, McAllister D, Vonderhaar EP, Palen K, Riese MJ, Gershan J, Johnson BD, Dwinell MB. STING agonist inflames the pancreatic cancer immune microenvironment and reduces tumor burden in mouse models. J Immunother Cancer. 2019;7(1):115. 31036082 10.1186/s40425-019-0573-5 PMC6489306

[50] Shi Y, Zheng W, Yang K, Harris KG, Ni K, Xue L, Lin W, Chang EB, Weichselbaum RR, Fu YX. Intratumoral accumulation of gut microbiota facilitates CD47-based immunotherapy via STING signaling. J Exp Med. 2020;217(5): Article e20192282. 32142585 10.1084/jem.20192282 PMC7201921

[51] Si W, Liang H, Bugno J, Xu Q, Ding X, Yang K, Fu Y, Weichselbaum RR, Zhao X, Wang L. *Lactobacillus rhamnosus* GG induces cGAS/STING- dependent type I interferon and improves response to immune checkpoint blockade. Gut. 2022;71(3):521–533. 33685966 10.1136/gutjnl-2020-323426 PMC8710942

[52] Kostic AD, Chun E, Robertson L, Glickman JN, Gallini CA, Michaud M, Clancy TE, Chung DC, Lochhead P, Hold GL, et al. *Fusobacterium nucleatum* potentiates intestinal tumorigenesis and modulates the tumor-immune microenvironment. Cell Host Microbe. 2013;14(2):207–215. 23954159 10.1016/j.chom.2013.07.007 PMC3772512

[53] Liu R, Wang J, Liu Y, Gao Y, Yang R. Regulation of gut microbiota on immune cell ferroptosis: A novel insight for immunotherapy against tumor. Cancer Lett. 2024;598: Article 217115. 39025428 10.1016/j.canlet.2024.217115

[54] Montalban-Arques A, Katkeviciute E, Busenhart P, Bircher A, Wirbel J, Zeller G, Morsy Y, Borsig L, Glaus Garzon JF, Müller A, et al. Commensal Clostridiales strains mediate effective anti-cancer immune response against solid tumors. Cell Host Microbe. 2021;29(10):1573–1588. 34453895 10.1016/j.chom.2021.08.001

[55] Overacre-Delgoffe AE, Bumgarner HJ, Cillo AR, Burr AHP, Tometich JT, Bhattacharjee A, Bruno TC, Vignali DAA, Hand TW. Microbiota-specific T follicular helper cells drive tertiary lymphoid structures and anti-tumor immunity against colorectal cancer. Immunity. 2021;54(12):2812–2824. 34861182 10.1016/j.immuni.2021.11.003 PMC8865366

[56] Xie Q, Ding J, Chen Y. Role of CD8^+^ T lymphocyte cells: Interplay with stromal cells in tumor microenvironment. Acta Pharm Sin B. 2021;11(6):1365–1378. 34221857 10.1016/j.apsb.2021.03.027 PMC8245853

[57] Zhu G, Su H, Johnson CH, Khan SA, Kluger H, Lu L. Intratumour microbiome associated with the infiltration of cytotoxic CD8^+^ T cells and patient survival in cutaneous melanoma. Eur J Cancer. 2021;151:25–34. 33962358 10.1016/j.ejca.2021.03.053 PMC8184628

[58] Owens JA, Saeedi BJ, Naudin CR, Hunter-Chang S, Barbian ME, Eboka RU, Askew L, Darby TM, Robinson BS, Jones RM. *Lactobacillus rhamnosus* GG orchestrates an antitumor immune response. Cell Mol Gastroenterol Hepatol. 2021;12(4):1311–1327. 34111601 10.1016/j.jcmgh.2021.06.001 PMC8463873

[59] Tanoue T, Morita S, Plichta DR, Skelly AN, Suda W, Sugiura Y, Narushima S, Vlamakis H, Motoo I, Sugita K, et al. A defined commensal consortium elicits CD8 T cells and anti-cancer immunity. Nature. 2019;565(7741):600–605. 30675064 10.1038/s41586-019-0878-z

[60] Yu AI, Zhao L, Eaton KA, Ho S, Chen J, Poe S, Becker J, Gonzalez A, McKinstry D, Hasso M, et al. Gut microbiota modulate CD8 T cell responses to influence colitis-associated tumorigenesis. Cell Rep. 2020;31(1): Article 107471. 32268087 10.1016/j.celrep.2020.03.035 PMC7934571

[61] Kreiter S, Vormehr M, van de Roemer N, Diken M, Löwer M, Diekmann J, Boegel S, Schrörs B, Vascotto F, Castle JC, et al. Mutant MHC class II epitopes drive therapeutic immune responses to cancer. Nature. 2015;520(7549):692–696. 25901682 10.1038/nature14426 PMC4838069

[62] Sahin U, Derhovanessian E, Miller M, Kloke BP, Simon P, Löwer M, Bukur V, Tadmor AD, Luxemburger U, Schrörs B, et al. Personalized RNA mutanome vaccines mobilize poly-specific therapeutic immunity against cancer. Nature. 2017;547(7662):222–226. 28678784 10.1038/nature23003

[63] Ott PA, Hu Z, Keskin DB, Shukla SA, Sun J, Bozym DJ, Zhang W, Luoma A, Giobbie-Hurder A, Peter L, et al. An immunogenic personal neoantigen vaccine for patients with melanoma. Nature. 2017;547(7662):217–221. 28678778 10.1038/nature22991 PMC5577644

[64] Wculek SK, Cueto FJ, Mujal AM, Melero I, Krummel MF, Sancho D. Dendritic cells in cancer immunology and immunotherapy. Nat Rev Immunol. 2020;20(1):7–24. 31467405 10.1038/s41577-019-0210-z

[65] Schaupp L, Muth S, Rogell L, Kofoed-Branzk M, Melchior F, Lienenklaus S, Ganal-Vonarburg SC, Klein M, Guendel F, Hain T, et al. Microbiota-induced type I interferons instruct a poised basal state of dendritic cells. Cell. 2020;181(5):1080–1096. 32380006 10.1016/j.cell.2020.04.022

[66] Böttcher JP, Bonavita E, Chakravarty P, Blees H, Cabeza-Cabrerizo M, Sammicheli S, Rogers NC, Sahai E, Zelenay S, Reis e Sousa C. NK cells stimulate recruitment of cDC1 into the tumor microenvironment promoting cancer immune control. Cell. 2018;172(5):1022–1037. 29429633 10.1016/j.cell.2018.01.004 PMC5847168

[67] Qiu Y, Jiang Z, Hu S, Wang L, Ma X, Yang X. *Lactobacillus plantarum* enhanced IL-22 production in natural killer (NK) cells that protect the integrity of intestinal epithelial cell barrier damaged by enterotoxigenic *Escherichia coli*. Int J Mol Sci. 2017;18(11): Article 2409. 29137183 10.3390/ijms18112409 PMC5713377

[68] Rizvi ZA, Dalal R, Sadhu S, Kumar Y, Kumar S, Gupta SK, Tripathy MR, Rathore DK, Awasthi A. High-salt diet mediates interplay between NK cells and gut microbiota to induce potent tumor immunity. Sci Adv. 2021;7(37):eabg5016. 34516769 10.1126/sciadv.abg5016 PMC8442882

[69] Vicente-Dueñas C, Janssen S, Oldenburg M, Auer F, González-Herrero I, Casado-García A, Isidro-Hernández M, Raboso-Gallego J, Westhoff P, Pandyra AA, et al. An intact gut microbiome protects genetically predisposed mice against leukemia. Blood. 2020;136(18):2003–2017. 32911536 10.1182/blood.2019004381 PMC7694022

[70] Peled JU, Gomes ALC, Devlin SM, Littmann ER, Taur Y, Sung AD, Weber D, Hashimoto D, Slingerland AE, Slingerland JB, et al. Microbiota as predictor of mortality in allogeneic hematopoietic-cell transplantation. N Engl J Med. 2020;382(9):822–834. 32101664 10.1056/NEJMoa1900623 PMC7534690

[71] Schluter J, Peled JU, Taylor BP, Markey KA, Smith M, Taur Y, Niehus R, Staffas A, Dai A, Fontana E, et al. The gut microbiota is associated with immune cell dynamics in humans. Nature. 2020;588(7837):303–307. 33239790 10.1038/s41586-020-2971-8 PMC7725892

[72] Fischer JC, Bscheider M, Eisenkolb G, Lin CC, Wintges A, Otten V, Lindemans CA, Heidegger S, Rudelius M, Monette S, et al. RIG-I/MAVS and STING signaling promote gut integrity during irradiation- and immune-mediated tissue injury. Sci Transl Med. 2017;9(386): Article eaag2513. 28424327 10.1126/scitranslmed.aag2513 PMC5604790

[73] Zhao H, Wu L, Yan G, Chen Y, Zhou M, Wu Y, Li Y. Inflammation and tumor progression: Signaling pathways and targeted intervention. Signal Transduct Target Ther. 2021;6:263. 34248142 10.1038/s41392-021-00658-5 PMC8273155

[74] Rubinstein MR, Wang X, Liu W, Hao Y, Cai G, Han YW. Fusobacterium nucleatum promotes colorectal carcinogenesis by modulating E-cadherin/β-catenin signaling via its FadA adhesin. Cell Host Microbe. 2013;14(2):195–206. 23954158 10.1016/j.chom.2013.07.012 PMC3770529

[75] Gur C, Ibrahim Y, Isaacson B, Yamin R, Abed J, Gamliel M, Enk J, Bar-On Y, Stanietsky-Kaynan N, Coppenhagen-Glazer S, et al. Binding of the Fap2 protein of fusobacterium nucleatum to human inhibitory receptor TIGIT protects tumors from immune cell attack. Immunity. 2015;42(2):344–355. 25680274 10.1016/j.immuni.2015.01.010 PMC4361732

[76] Yue Y, Ye K, Lu J, Wang X, Zhang S, Liu L, Yang B, Nassar K, Xu X, Pang X, et al. Probiotic strain *Lactobacillus plantarum* YYC-3 prevents colon cancer in mice by regulating the tumour microenvironment. Biomed Pharmacother. 2020;127: Article 110159. 32353824 10.1016/j.biopha.2020.110159

[77] Nomura M, Nagatomo R, Doi K, Shimizu J, Baba K, Saito T, Matsumoto S, Inoue K, Muto M. Association of short-chain fatty acids in the gut microbiome with clinical response to treatment with nivolumab or pembrolizumab in patients with solid cancer tumors. JAMA Netw Open. 2020;3(4): Article e202895. 32297948 10.1001/jamanetworkopen.2020.2895 PMC7163404

[78] Zagato E, Pozzi C, Bertocchi A, Schioppa T, Saccheri F, Guglietta S, Fosso B, Melocchi L, Nizzoli G, Troisi J, et al. Endogenous murine microbiota member *Faecalibaculum rodentium* and its human homologue protect from intestinal tumour growth. Nat Microbiol. 2020;5(3):511–524. 31988379 10.1038/s41564-019-0649-5 PMC7048616

[79] Bachem A, Makhlouf C, Binger KJ, de Souza DP, Tull D, Hochheiser K, Whitney PG, Fernandez-Ruiz D, Dähling S, Kastenmüller W, et al. Microbiota-derived short-chain fatty acids promote the memory potential of antigen-activated CD8^+^ T cells. Immunity. 2019;51(2):285–297. 31272808 10.1016/j.immuni.2019.06.002

[80] He Y, Fu L, Li Y, Wang W, Gong M, Zhang J, Dong X, Huang J, Wang Q, Mackay CR, et al. Gut microbial metabolites facilitate anticancer therapy efficacy by modulating cytotoxic CD8^+^ T cell immunity. Cell Metab. 2021;33(5):988–1000. 33761313 10.1016/j.cmet.2021.03.002

[81] Luu M, Riester Z, Baldrich A, Reichardt N, Yuille S, Busetti A, Klein M, Wempe A, Leister H, Raifer H, et al. Microbial short-chain fatty acids modulate CD8^+^ T cell responses and improve adoptive immunotherapy for cancer. Nat Commun. 2021;12(1):4077. 34210970 10.1038/s41467-021-24331-1 PMC8249424

[82] Kim M, Qie Y, Park J, Kim CH. Gut microbial metabolites fuel host antibody responses. Cell Host Microbe. 2016;20(2):202–214. 27476413 10.1016/j.chom.2016.07.001 PMC4982788

[83] Botticelli A, Vernocchi P, Marini F, Quagliariello A, Cerbelli B, Reddel S, del Chierico F, di Pietro F, Giusti R, Tomassini A, et al. Gut metabolomics profiling of non-small cell lung cancer (NSCLC) patients under immunotherapy treatment. J Transl Med. 2020;18(1):49. 32014010 10.1186/s12967-020-02231-0 PMC6998840

[84] Coutzac C, Jouniaux J, Paci A, Schmidt J, Mallardo D, Seck A, Asvatourian V, Cassard L, Saulnier P, Lacroix L, et al. Systemic short chain fatty acids limit antitumor effect of CTLA-4 blockade in hosts with cancer. Nat Commun. 2020;11(1):2168. 32358520 10.1038/s41467-020-16079-x PMC7195489

[85] Kim IS, Jo E. Inosine: A bioactive metabolite with multimodal actions in human diseases. Front Pharmacol. 2022;13:1043970. 36467085 10.3389/fphar.2022.1043970 PMC9708727

[86] Wang T, Gnanaprakasam JNR, Chen X, Kang S, Xu X, Sun H, Liu L, Rodgers H, Miller E, Cassel TA, et al. Inosine is an alternative carbon source for CD8^+^-T-cell function under glucose restriction. Nat Metab. 2020;2(7):635–647. 32694789 10.1038/s42255-020-0219-4 PMC7371628

[87] Mager LF, Burkhard R, Pett N, Cooke NCA, Brown K, Ramay H, Paik S, Stagg J, Groves RA, Gallo M, et al. Microbiome-derived inosine modulates response to checkpoint inhibitor immunotherapy. Science. 2020;369(6510):1481–1489. 32792462 10.1126/science.abc3421

[88] Zhang L, Jiang L, Yu L, Li Q, Tian X, He J, Zeng L, Yang Y, Wang C, Wei Y, et al. Inhibition of UBA6 by inosine augments tumour immunogenicity and responses. Nat Commun. 2022;13(1):5413. 36109526 10.1038/s41467-022-33116-z PMC9478149

[89] Paik D, Yao L, Zhang Y, Bae S, D’Agostino GD, Zhang M, Kim E, Franzosa EA, Avila-Pacheco J, Bisanz JE, et al. Human gut bacteria produce Τ(Η)17-modulating bile acid metabolites. Nature. 2022;603(7903):907–912. 35296854 10.1038/s41586-022-04480-z PMC9132548

[90] Campbell C, McKenney PT, Konstantinovsky D, Isaeva OI, Schizas M, Verter J, Mai C, Jin WB, Guo CJ, Violante S, et al. Bacterial metabolism of bile acids promotes generation of peripheral regulatory T cells. Nature. 2020;581(7809):475–479. 32461639 10.1038/s41586-020-2193-0 PMC7540721

[91] Ma C, Han M, Heinrich B, Fu Q, Zhang Q, Sandhu M, Agdashian D, Terabe M, Berzofsky JA, Fako V, et al. Gut microbiome-mediated bile acid metabolism regulates liver cancer via NKT cells. Science. 2018;360(6391): Article eaan5931. 29798856 10.1126/science.aan5931 PMC6407885

[92] Mezrich JD, Fechner JH, Zhang X, Johnson BP, Burlingham WJ, Bradfield CA. An interaction between kynurenine and the aryl hydrocarbon receptor can generate regulatory T cells. J Immunol. 2010(185, 6):3190–3198. 10.4049/jimmunol.0903670 PMC2952546 20720200

[93] Li H, Bullock K, Gurjao C, Braun D, Shukla SA, Bossé D, Lalani AKA, Gopal S, Jin C, Horak C, et al. Metabolomic adaptations and correlates of survival to immune checkpoint blockade. Nat Commun. 2019;10:4346. 31554815 10.1038/s41467-019-12361-9 PMC6761178

[94] Kocher F, Amann A, Zimmer K, Geisler S, Fuchs D, Pichler R, Wolf D, Kurz K, Seeber A, Pircher A. High indoleamine-2,3-dioxygenase 1 (IDO) activity is linked to primary resistance to immunotherapy in non-small cell lung cancer (NSCLC). Transl Lung Cancer Res. 2021;10(1):304–313. 33569314 10.21037/tlcr-20-380 PMC7867793

[95] Liu Y, Zhou N, Zhou L, Wang J, Zhou Y, Zhang T, Fang Y, Deng J, Gao Y, Liang X, et al. IL-2 regulates tumor-reactive CD8^+^ T cell exhaustion by activating the aryl hydrocarbon receptor. Nat Immunol. 2021;22(3):358–369. 33432230 10.1038/s41590-020-00850-9

[96] Limeta A, Ji B, Levin M, Gatto F, Nielsen J. Meta-analysis of the gut microbiota in predicting response to cancer immunotherapy in metastatic melanoma. JCI Insight. 2020;5(23): Article e140940. 33268597 10.1172/jci.insight.140940 PMC7714408

[97] Wang H, Rong X, Zhao G, Zhou Y, Xiao Y, Ma D, Jin X, Wu Y, Yan Y, Yang H, et al. The microbial metabolite trimethylamine N-oxide promotes antitumor immunity in triple-negative breast cancer. Cell Metab. 2022;34(4):581–594. 35278352 10.1016/j.cmet.2022.02.010

[98] Mirji G, Worth A, Bhat SA, el Sayed M, Kannan T, Goldman AR, Tang HY, Liu Q, Auslander N, Dang CV, et al. The microbiome-derived metabolite TMAO drives immune activation and boosts responses to immune checkpoint blockade in pancreatic cancer. Sci Immunol. 2022;7(75):eabn0704. 36083892 10.1126/sciimmunol.abn0704 PMC9925043

[99] Usyk M, Pandey A, Hayes RB, Moran U, Pavlick A, Osman I, Weber JS, Ahn J. *Bacteroides vulgatus* and *Bacteroides dorei* predict immune-related adverse events in immune checkpoint blockade treatment of metastatic melanoma. Genome Med. 2021;13(1):160. 34641962 10.1186/s13073-021-00974-z PMC8513370

[100] Andrews MC, Duong CPM, Gopalakrishnan V, Iebba V, Chen WS, Derosa L, Khan MAW, Cogdill AP, White MG, Wong MC, et al. Gut microbiota signatures are associated with toxicity to combined CTLA-4 and PD-1 blockade. Nat Med. 2021;27:1432–1441. 34239137 10.1038/s41591-021-01406-6 PMC11107795

[101] Sun S, Luo L, Liang W, Yin Q, Guo J, Rush AM, Lv Z, Liang Q, Fischbach MA, Sonnenburg JL, et al. Bifidobacterium alters the gut microbiota and modulates the functional metabolism of T regulatory cells in the context of immune checkpoint blockade. Proc Natl Acad Sci USA. 2020;117(44):27509–27515. 33077598 10.1073/pnas.1921223117 PMC7959554

[102] Wang F, Yin Q, Chen L, Davis MM. Bifidobacterium can mitigate intestinal immunopathology in the context of CTLA-4 blockade. Proc Natl Acad Sci USA. 2018;115(1):157–161. 29255057 10.1073/pnas.1712901115 PMC5776803

[103] Galluzzi L, Guilbaud E, Schmidt D, Kroemer G, Marincola FM. Targeting immunogenic cell stress and death for cancer therapy. Nat Rev Drug Discov. 2024;23:445–460. 38622310 10.1038/s41573-024-00920-9 PMC11153000

[104] Uribe-Herranz M, Rafail S, Beghi S, Gil-de-Gómez L, Verginadis I, Bittinger K, Pustylnikov S, Pierini S, Perales-Linares R, Blair IA, et al. Gut microbiota modulate dendritic cell antigen presentation and radiotherapy-induced antitumor immune response. J Clin Invest. 2020;130(1):466–479. 31815742 10.1172/JCI124332 PMC6934221

[105] Li L, He S, Liao B, Wang M, Lin H, Hu B, Lan X, Shu Z, Zhang C, Yu M, et al. Orally administrated hydrogel harnessing intratumoral microbiome and microbiota-related immune responses for potentiated colorectal cancer treatment. Research. 2024;7:364. 10.34133/research.0364 PMC11077293 38721274

[106] Viaud S, Saccheri F, Mignot G, Yamazaki T, Daillère R, Hannani D, Enot DP, Pfirschke C, Engblom C, Pittet MJ, et al. The intestinal microbiota modulates the anticancer immune effects of cyclophosphamide. Science. 2013;342(6161):971–976. 24264990 10.1126/science.1240537 PMC4048947

[107] Bowers JS, Nelson MH, Kundimi S, Bailey SR, Huff LW, Schwartz KM, Cole DJ, Rubinstein MP, Paulos CM. Dendritic cells in irradiated mice trigger the functional plasticity and antitumor activity of adoptively transferred Tc17 cells via IL12 signaling. Clin Cancer Res. 2015;21(11):2546–2557. 25904754 10.1158/1078-0432.CCR-14-2294 PMC4452402

[108] Woese CR, Fox GE. Phylogenetic structure of the prokaryotic domain: The primary kingdoms. Proc Natl Acad Sci USA. 1977;74(11):5088–5090. 270744 10.1073/pnas.74.11.5088 PMC432104

[109] Schwartz DJ, Rebeck ON, Dantas G. Complex interactions between the microbiome and cancer immune therapy. Crit Rev Clin Lab Sci. 2019;56(8):567–585. 31526274 10.1080/10408363.2019.1660303 PMC6776419

[110] Fu A, Yao B, Dong T, Chen Y, Yao J, Liu Y, Li H, Bai H, Liu X, Zhang Y, et al. Tumor-resident intracellular microbiota promotes metastatic colonization in breast cancer. Cell. 2022;185(8):1356–1372. 35395179 10.1016/j.cell.2022.02.027

[111] Quince C, Walker AW, Simpson JT, Loman NJ, Segata N. Shotgun metagenomics, from sampling to analysis. Nat Biotechnol. 2017;35(9):833–844. 28898207 10.1038/nbt.3935

[112] Cullin N, Azevedo Antunes C, Straussman R, Stein-Thoeringer CK, Elinav E. Microbiome and cancer. Cancer Cell. 2021;39:1317–1341. 34506740 10.1016/j.ccell.2021.08.006

[113] Kuchina A, Brettner LM, Paleologu L, Roco CM, Rosenberg AB, Carignano A, Kibler R, Hirano M, DePaolo RW, Seelig G. Microbial single-cell RNA sequencing by split-pool barcoding. Science. 2021;371(6531): Article eaba5257. 33335020 10.1126/science.aba5257 PMC8269303

[114] Avital G, Avraham R, Fan A, Hashimshony T, Hung DT, Yanai I. scDual-Seq: Mapping the gene regulatory program of salmonella infection by host and pathogen single-cell RNA-sequencing. Genome Biol. 2017;18(1):200. 29073931 10.1186/s13059-017-1340-x PMC5658913

[115] Blattman SB, Jiang W, Oikonomou P, Tavazoie S. Prokaryotic single-cell RNA sequencing by in situ combinatorial indexing. Nat Microbiol. 2020;5(10):1192–1201. 32451472 10.1038/s41564-020-0729-6 PMC8330242

[116] Dar D, Dar N, Cai L, Newman DK. Spatial transcriptomics of planktonic and sessile bacterial populations at single-cell resolution. Science. 2021;373(6556): Article eabi4882. 34385369 10.1126/science.abi4882 PMC8454218

[117] Shi H, Shi Q, Grodner B, Lenz JS, Zipfel WR, Brito IL, de Vlaminck I. Highly multiplexed spatial mapping of microbial communities. Nature. 2020;588:676–681. 33268897 10.1038/s41586-020-2983-4 PMC8050837

[118] Rao A, Barkley D, França GS, Yanai I. Exploring tissue architecture using spatial transcriptomics. Nature. 2021;596:211–220. 34381231 10.1038/s41586-021-03634-9 PMC8475179

[119] Galeano Niño JL, Wu H, LaCourse KD, Kempchinsky AG, Baryiames A, Barber B, Futran N, Houlton J, Sather C, Sicinska E, et al. Effect of the intratumoral microbiota on spatial and cellular heterogeneity in cancer. Nature. 2022;611(7937):810–817. 36385528 10.1038/s41586-022-05435-0 PMC9684076

[120] Kalaora S, Nagler A, Nejman D, Alon M, Barbolin C, Barnea E, Ketelaars SLC, Cheng K, Vervier K, Shental N, et al. Identification of bacteria-derived HLA-bound peptides in melanoma. Nature. 2021;592(7852):138–143. 33731925 10.1038/s41586-021-03368-8 PMC9717498

[121] Maier L, Pruteanu M, Kuhn M, Zeller G, Telzerow A, Anderson EE, Brochado AR, Fernandez KC, Dose H, Mori H, et al. Extensive impact of non-antibiotic drugs on human gut bacteria. Nature. 2018;555(7698):623–628. 29555994 10.1038/nature25979 PMC6108420

[122] Davar D, Dzutsev AK, McCulloch JA, Rodrigues RR, Chauvin JM, Morrison RM, Deblasio RN, Menna C, Ding Q, Pagliano O, et al. Fecal microbiota transplant overcomes resistance to anti-PD-1 therapy in melanoma patients. Science. 2021;371(6529):595–602. 33542131 10.1126/science.abf3363 PMC8097968

[123] Baruch EN, Youngster I, Ben-Betzalel G, Ortenberg R, Lahat A, Katz L, Adler K, Dick-Necula D, Raskin S, Bloch N, et al. Fecal microbiota transplant promotes response in immunotherapy-refractory melanoma patients. Science. 2021;371(6529):602–609. 33303685 10.1126/science.abb5920

[124] Zhu C, Sawrey-Kubicek L, Beals E, Rhodes CH, Houts HE, Sacchi R, Zivkovic AM. Human gut microbiome composition and tryptophan metabolites were changed differently by fast food and Mediterranean diet in 4 days: A pilot study. Nutr Res. 2020;77:62–72. 32330749 10.1016/j.nutres.2020.03.005

[125] David LA, Maurice CF, Carmody RN, Gootenberg DB, Button JE, Wolfe BE, Ling AV, Devlin AS, Varma Y, Fischbach MA, et al. Diet rapidly and reproducibly alters the human gut microbiome. Nature. 2014;505:559–563. 24336217 10.1038/nature12820 PMC3957428

[126] Simpson RC, Shanahan ER, Batten M, Reijers ILM, Read M, Silva IP, Versluis JM, Ribeiro R, Angelatos AS, Tan J, et al. Diet-driven microbial ecology underpins associations between cancer immunotherapy outcomes and the gut microbiome. Nat Med. 2022;28:2344–2352. 36138151 10.1038/s41591-022-01965-2

[127] Spencer CN, McQuade JL, Gopalakrishnan V, McCulloch JA, Vetizou M, Cogdill AP, Khan MAW, Zhang X, White MG, Peterson CB, et al. Dietary fiber and probiotics influence the gut microbiome and melanoma immunotherapy response. Science. 2021;374(6575):1632–1640. 34941392 10.1126/science.aaz7015 PMC8970537

[128] Ferrere G, Tidjani Alou M, Liu P, Goubet AG, Fidelle M, Kepp O, Durand S, Iebba V, Fluckiger A, Daillère R, et al. Ketogenic diet and ketone bodies enhance the anticancer effects of PD-1 blockade. JCI Insight. 2021;6(2): Article e145207. 33320838 10.1172/jci.insight.145207 PMC7934884

[129] Han K, Nam J, Xu J, Sun X, Huang X, Animasahun O, Achreja A, Jeon JH, Pursley B, Kamada N, et al. Generation of systemic antitumour immunity via the in situ modulation of the gut microbiome by an orally administered inulin gel. Nat Biomed Eng. 2021;5(11):1377–1388. 34168321 10.1038/s41551-021-00749-2 PMC8595497

[130] Zhang S, Mao Y, Zhang Z, Li ZM, Kong CY, Chen HL, Cai PR, Han B, Ye T, Wang LS. Pectin supplement significantly enhanced the anti-PD-1 efficacy in tumor-bearing mice humanized with gut microbiota from patients with colorectal cancer. Theranostics. 2021;11(9):4155–4170. 33754054 10.7150/thno.54476 PMC7977465

[131] Riaz Rajoka MS, Zhao H, Lu Y, Lian Z, Li N, Hussain N, Shao D, Jin M, Li Q, Shi J. Anticancer potential against cervix cancer (HeLa) cell line of probiotic lactobacillus casei and *Lactobacillus paracasei* strains isolated from human breast milk. Food Funct. 2018;9(5):2705–2715. 29762617 10.1039/c8fo00547h

[132] Cheng K, Zhao R, Li Y, Qi Y, Wang Y, Zhang Y, Qin H, Qin Y, Chen L, Li C, et al. Bioengineered bacteria-derived outer membrane vesicles as a versatile antigen display platform for tumor vaccination via plug-and-display technology. Nat Commun. 2021;12(1):2041. 33824314 10.1038/s41467-021-22308-8 PMC8024398

[133] Zhang S, Han B, Mao Y, Zhang ZY, Li ZM, Kong CY, Wu Y, Chen GQ, Wang LS. *Lacticaseibacillus paracasei sh2020* induced antitumor immunity and synergized with anti-programmed cell death 1 to reduce tumor burden in mice. Gut Microbes. 2022;14(1):2046246. 35259052 10.1080/19490976.2022.2046246 PMC8920197

[134] Dizman N, Meza L, Bergerot P, Alcantara M, Dorff T, Lyou Y, Frankel P, Cui Y, Mira V, Llamas M, et al. Nivolumab plus ipilimumab with or without live bacterial supplementation in metastatic renal cell carcinoma: A randomized phase 1 trial. Nat Med. 2022;28:704–712. 35228755 10.1038/s41591-022-01694-6 PMC9018425

[135] Zmora N, Zilberman-Schapira G, Suez J, Mor U, Dori-Bachash M, Bashiardes S, Kotler E, Zur M, Regev-Lehavi D, Brik RBZ, et al. Personalized gut mucosal colonization resistance to empiric probiotics is associated with unique host and microbiome features. Cell. 2018;174(6):1388–1405. 30193112 10.1016/j.cell.2018.08.041

[136] Veiga P, Suez J, Derrien M, Elinav E. Moving from probiotics to precision probiotics. Nat Microbiol. 2020;5:878–880. 32393856 10.1038/s41564-020-0721-1

[137] Liou J, Malfertheiner P, Smith SI, El-Omar EM, Wu M. 40 years after the discovery of *Helicobacter pylori*: Towards elimination of H pylori for gastric cancer prevention. Lancet. 2024;403(10444):2570–2572. 38879242 10.1016/S0140-6736(24)01171-1

[138] Hattori N, Niwa T, Ishida T, Kobayashi K, Imai T, Mori A, Kimura K, Mori T, Asami Y, Ushijima T. Antibiotics suppress colon tumorigenesis through inhibition of aberrant DNA methylation in an azoxymethane and dextran sulfate sodium colitis model. Cancer Sci. 2019;110(1):147–156. 30443963 10.1111/cas.13880 PMC6317928

[139] Cao H, Xu M, Dong W, Deng B, Wang S, Zhang Y, Wang S, Luo S, Wang W, Qi Y, et al. Secondary bile acid-induced dysbiosis promotes intestinal carcinogenesis. Int J Cancer. 2017;140(11):2545–2556. 28187526 10.1002/ijc.30643

[140] Pinato DJ, Howlett S, Ottaviani D, Urus H, Patel A, Mineo T, Brock C, Power D, Hatcher O, Falconer A, et al. Association of prior antibiotic treatment with survival and response to immune checkpoint inhibitor therapy in patients with cancer. JAMA Oncol. 2019;5(12):1774–1778. 31513236 10.1001/jamaoncol.2019.2785 PMC6743060

[141] Derosa L, Routy B, Fidelle M, Iebba V, Alla L, Pasolli E, Segata N, Desnoyer A, Pietrantonio F, Ferrere G, et al. Gut bacteria composition drives primary resistance to cancer immunotherapy in renal cell carcinoma patients. Eur Urol. 2020;78(2):195–206. 32376136 10.1016/j.eururo.2020.04.044

[142] Zhao L, Li Y, Jiang N, Song X, Xu J, Zhu X, Chen C, Kong C, Wang X, Zong D, et al. Association of blood biochemical indexes and antibiotic exposure with severe immune-related adverse events in patients with advanced cancers receiving PD-1 inhibitors. J Immunother. 2022;45(4):210–216. 35250004 10.1097/CJI.0000000000000415 PMC8986630

[143] Vehreschild MJGT, Ducher A, Louie T, Cornely OA, Feger C, Dane A, Varastet M, Vitry F, de Gunzburg J, Andremont A, et al. An open randomized multicentre phase 2 trial to assess the safety of DAV132 and its efficacy to protect gut microbiota diversity in hospitalized patients treated with fluoroquinolones. J Antimicrob Chemother. 2022;77(4):1155–1165. 35016205 10.1093/jac/dkab474 PMC8969469

[144] de Gunzburg J, Ghozlane A, Ducher A, le Chatelier E, Duval X, Ruppé E, Armand-Lefevre L, Sablier-Gallis F, Burdet C, Alavoine L, et al. Protection of the human gut microbiome from antibiotics. J Infect Dis. 2018;217(4):628–636. 29186529 10.1093/infdis/jix604 PMC5853327

[145] Zheng D, Dong X, Pan P, Chen KW, Fan JX, Cheng SX, Zhang XZ. Phage-guided modulation of the gut microbiota of mouse models of colorectal cancer augments their responses to chemotherapy. Nat Biomed Eng. 2019;3:717–728. 31332342 10.1038/s41551-019-0423-2

[146] Lynch JP, Goers L, Lesser CF. Emerging strategies for engineering *Escherichia coli* Nissle 1917-based therapeutics. Trends Pharmacol Sci. 2022;43(9):772–786. 35232591 10.1016/j.tips.2022.02.002 PMC9378478

[147] Leventhal DS, Sokolovska A, Li N, Plescia C, Kolodziej SA, Gallant CW, Christmas R, Gao JR, James MJ, Abin-Fuentes A, et al. Immunotherapy with engineered bacteria by targeting the STING pathway for anti-tumor immunity. Nat Commun. 2020;11(1):2739. 32483165 10.1038/s41467-020-16602-0 PMC7264239

[148] Griffin ME, Espinosa J, Becker JL, Luo JD, Carroll TS, Jha JK, Fanger GR, Hang HC. Enterococcus peptidoglycan remodeling promotes checkpoint inhibitor cancer immunotherapy. Science. 2021;373(6558):1040–1046. 34446607 10.1126/science.abc9113 PMC9503018

[149] Din MO, Danino T, Prindle A, Skalak M, Selimkhanov J, Allen K, Julio E, Atolia E, Tsimring LS, Bhatia SN, et al. Synchronized cycles of bacterial lysis for in vivo delivery. Nature. 2016;536(7614):81–85. 27437587 10.1038/nature18930 PMC5048415

[150] Gurbatri CR, Lia I, Vincent R, Coker C, Castro S, Treuting PM, Hinchliffe TE, Arpaia N, Danino T. Engineered probiotics for local tumor delivery of checkpoint blockade nanobodies. Sci Transl Med. 2020;12(530): Article eaax0876. 32051224 10.1126/scitranslmed.aax0876 PMC7685004

[151] Canale FP, Basso C, Antonini G, Perotti M, Li N, Sokolovska A, Neumann J, James MJ, Geiger S, Jin W, et al. Metabolic modulation of tumours with engineered bacteria for immunotherapy. Nature. 2021;598:662–666. 34616044 10.1038/s41586-021-04003-2

[152] Geiger R, Rieckmann JC, Wolf T, Basso C, Feng Y, Fuhrer T, Kogadeeva M, Picotti P, Meissner F, Mann M, et al. L-arginine modulates T cell metabolism and enhances survival and anti-tumor activity. Cell. 2016;167(3):829–842. 27745970 10.1016/j.cell.2016.09.031 PMC5075284

[153] Kelly CM, Antonescu CR, Bowler T, Munhoz R, Chi P, Dickson MA, Gounder MM, Keohan ML, Movva S, Dholakia R, et al. Objective response rate among patients with locally advanced or metastatic sarcoma treated with talimogene laherparepvec in combination with pembrolizumab: A phase 2 clinical trial. JAMA Oncol. 2020;6:402–408. 31971541 10.1001/jamaoncol.2019.6152 PMC6990941

[154] Lang FF, Conrad C, Gomez-Manzano C, Yung WKA, Sawaya R, Weinberg JS, Prabhu SS, Rao G, Fuller GN, Aldape KD, et al. Phase I study of DNX-2401 (Delta-24-RGD) oncolytic adenovirus: Replication and immunotherapeutic effects in recurrent malignant glioma. J Clin Oncol. 2018;36(14):1419–1427. 29432077 10.1200/JCO.2017.75.8219 PMC6075856

[155] Janku F, Zhang HH, Pezeshki A, Goel S, Murthy R, Wang-Gillam A, Shepard DR, Helgason T, Masters T, Hong DS, et al. Intratumoral injection of clostridium novyi-NT spores in patients with treatment-refractory advanced solid tumors. Clin Cancer Res. 2021;27:96–106. 33046513 10.1158/1078-0432.CCR-20-2065

[156] Zhou S, Gravekamp C, Bermudes D, Liu K. Tumour-targeting bacteria engineered to fight cancer. Nat Rev Cancer. 2018;18:727–743. 30405213 10.1038/s41568-018-0070-z PMC6902869

[157] Zhu X, Li K, Liu G, Wu R, Zhang Y, Wang S, Xu M, Lu L, Li P. Microbial metabolite butyrate promotes anti-PD-1 antitumor efficacy by modulating T cell receptor signaling of cytotoxic CD8 T cell. Gut Microbes. 2023;15:2249143. 37635362 10.1080/19490976.2023.2249143 PMC10464552

[158] Kang X, Liu C, Ding Y, Ni Y, Ji F, Lau HCH, Jiang L, Sung JJY, Wong SH, Yu J. *Roseburia intestinalis* generated butyrate boosts anti-PD-1 efficacy in colorectal cancer by activating cytotoxic CD8^+^ T cells. Gut. 2023;72(11):2112–2122. 37491158 10.1136/gutjnl-2023-330291 PMC10579466

[159] Wei Y, Liu W, Wang R, Chen Y, Liu J, Guo X, Can C, Yang X, Wang D, Hu X, et al. Propionate promotes ferroptosis and apoptosis through mitophagy and ACSL4-mediated ferroptosis elicits anti-leukemia immunity. Free Radic Biol Med. 2024;213:36–51. 38215892 10.1016/j.freeradbiomed.2024.01.005

[160] Liu Y, Zhou Q, Ye F, Yang C, Jiang H. Gut microbiota-derived short-chain fatty acids promote prostate cancer progression via inducing cancer cell autophagy and M2 macrophage polarization. Neoplasia. 2023;43: Article 100928. 37579688 10.1016/j.neo.2023.100928 PMC10429288

[161] Miller KD, O'Connor S, Pniewski KA, Kannan T, Acosta R, Mirji G, Papp S, Hulse M, Mukha D, Hlavaty S, et al. Acetate acts as a metabolic immunomodulator by bolstering T-cell effector function and potentiating antitumor immunity in breast cancer. Nat Cancer. 2023;4(10):1491–1507. 37723305 10.1038/s43018-023-00636-6 PMC10615731

[162] Trompette A, Gollwitzer ES, Pattaroni C, Lopez-Mejia IC, Riva E, Pernot J, Ubags N, Fajas L, Nicod LP, Marsland BJ. Dietary fiber confers protection against flu by shaping Ly6c^-^ patrolling monocyte hematopoiesis and CD8^+^ T cell metabolism. Immunity. 2018;48(5):992–1005. 29768180 10.1016/j.immuni.2018.04.022

[163] Mowat C, Dhatt J, Bhatti I, Hamie A, Baker K. Short chain fatty acids prime colorectal cancer cells to activate antitumor immunity. Front Immunol. 2023;14:1190810. 37304266 10.3389/fimmu.2023.1190810 PMC10248408

[164] Cong J, Liu P, Han Z, Ying W, Li C, Yang Y, Wang S, Yang J, Cao F, Shen J, et al. Bile acids modified by the intestinal microbiota promote colorectal cancer growth by suppressing CD8^+^ T cell effector functions. Immunity. 2024;57(4):876–889. 38479384 10.1016/j.immuni.2024.02.014

[165] Hezaveh K, Shinde RS, Klötgen A, Halaby MJ, Lamorte S, Ciudad MT, Quevedo R, Neufeld L, Liu ZQ, Jin R, et al. Tryptophan-derived microbial metabolites activate the aryl hydrocarbon receptor in tumor-associated macrophages to suppress anti-tumor immunity. Immunity. 2022;55:324–340. 35139353 10.1016/j.immuni.2022.01.006 PMC8888129

[166] Bender MJ, McPherson AC, Phelps CM, Pandey SP, Laughlin CR, Shapira JH, Medina Sanchez L, Rana M, Richie TG, Mims TS, et al. Dietary tryptophan metabolite released by intratumoral *Lactobacillus reuteri* facilitates immune checkpoint inhibitor treatment. Cell. 2023;186(9):1846–1862. 37028428 10.1016/j.cell.2023.03.011 PMC10148916

[167] Jia D, Wang Q, Qi Y, Jiang Y, He J, Lin Y, Sun Y, Xu J, Chen W, Fan L, et al. Microbial metabolite enhances immunotherapy efficacy by modulating T cell stemness in pan-cancer. Cell. 2024;187(7):1651–1665. 38490195 10.1016/j.cell.2024.02.022

